# Glycan-Based Flow-Through Device for the Detection of SARS-COV-2

**DOI:** 10.1021/acssensors.1c01470

**Published:** 2021-10-11

**Authors:** Alexander N. Baker, Sarah-Jane Richards, Sarojini Pandey, Collette S. Guy, Ashfaq Ahmad, Muhammad Hasan, Caroline I. Biggs, Panagiotis G. Georgiou, Alexander J. Zwetsloot, Anne Straube, Simone Dedola, Robert A. Field, Neil R. Anderson, Marc Walker, Dimitris Grammatopoulos, Matthew I. Gibson

**Affiliations:** † Department of Chemistry, University of Warwick, Coventry CV4 7AL, U.K.; ‡ School of Life Sciences, University of Warwick, Coventry CV4 7AL, U.K.; § Warwick Medical School, University of Warwick, Coventry CV4 7AL, U.K.; ∥ Department of Physics, University of Warwick, Coventry CV4 7AL, U.K.; ⊥ Institute of Precision Diagnostics and Translational Medicine, University Hospitals Coventry and Warwickshire NHS Trust, Clifford Bridge Road, Coventry CV2 2DX, U.K.; # Iceni Diagnostics Ltd., Norwich Research Park, Norwich NR4 7GJ, U.K.; ∇ Department of Chemistry and Manchester Institute of Biotechnology, University of Manchester, Manchester M1 7DN, U.K.

**Keywords:** COVID-19, SARS-COV-2, glycans, nanoparticles, polymers, lateral flow, flow-through, rapid diagnostics, glycobiology

## Abstract

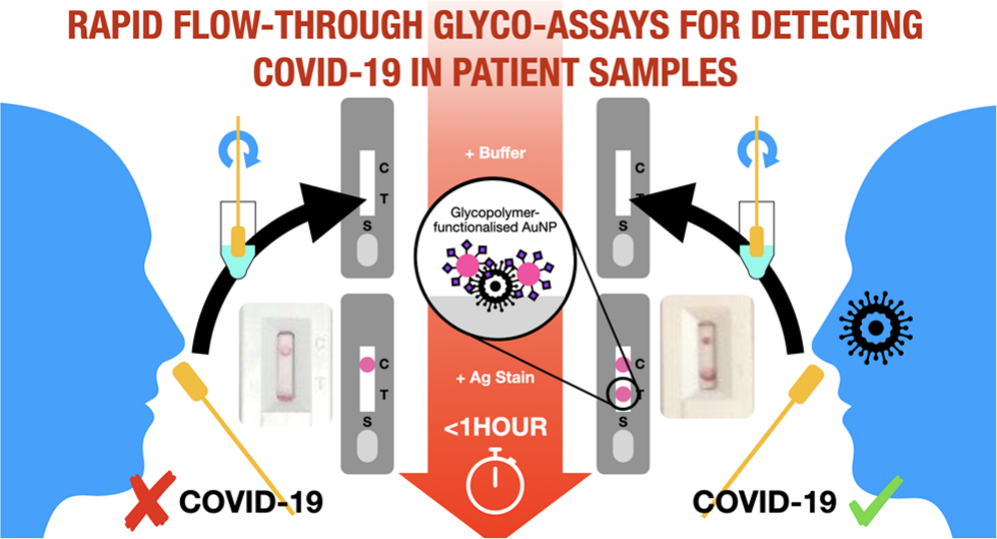

The COVID-19 pandemic, and future pandemics, require diagnostic tools to track disease spread and guide the isolation of (a)symptomatic individuals. Lateral-flow diagnostics (LFDs) are rapid and of lower cost than molecular (genetic) tests, with current LFDs using antibodies as their recognition units. Herein, we develop a prototype flow-through device (related, but distinct to LFDs), utilizing *N-*acetyl neuraminic acid-functionalized, polymer-coated, gold nanoparticles as the detection/capture unit for SARS-COV-2, by targeting the sialic acid-binding site of the spike protein. The prototype device can give rapid results, with higher viral loads being faster than lower viral loads. The prototype’s effectiveness is demonstrated using spike protein, lentiviral models, and a panel of heat-inactivated primary patient nasal swabs. The device was also shown to retain detection capability toward recombinant spike proteins from several variants (mutants) of concern. This study provides the proof of principle that glyco-lateral-flow devices could be developed to be used in the tracking monitoring of infectious agents, to complement, or as alternatives to antibody-based systems.

The COVID-19 pandemic has led to >171 million confirmed cases and ∼3.7 million deaths worldwide, reported to WHO, as of the 4th of June 2021.^1^ COVID-19 is caused by the coronavirus SARS-COV-2, first reported in Wuhan (China).^2^ Despite global efforts, there are still a limited number of effective therapeutics. Vaccines have now been approved for use, but with limited supplies; a major mechanism for controlling disease spread remains testing, identification, and patient isolation.

The testing system deployed by more economically developed countries (MEDCs) and less economically developed countries (LEDCs) has been based primarily on molecular (genetic) approaches such as real-time reverse-transcription polymerase chain reaction (rRT-PCR).^3−6^ However, RT-PCR-based approaches require dedicated laboratory facilities and trained personnel, meaning early in the pandemic CT scans, which are not recommended for routine use, were initially employed.^7^ Due to the infrastructure needs of RT-PCR and long processing times, RT-PCR does not typically provide a rapid turnaround, especially in a high volume laboratory setting, although it is considered the gold standard for COVID-19 diagnosis. In July 2020 during the early stages of the COVID-19 pandemic, in the United States, the average wait time for an RT-PCR test result was 4 days with 37% of people receiving the results within 2 days.^8^ The availability of RT-PCR testing also varies significantly between countries; per 1000 people (31/7/2020)^9^ the United Kingdom (2.27) and the United States (2.91) have significantly out-tested LEDCs such as Zimbabwe (0.07) or Myanmar (0.01).^9^ In Iran, for example, CT scanners are more abundant^10^ than RT-PCR machines.^11,12^ Faster RT-PCR devices, such as those based on DNAnudge, have been developed and allow for decentralized testing outside of hospital or lab environments but do have capacity requirements of one machine to one test.^13^ Other molecular genetic techniques have also been developed, which similarly do not require centralized testing infrastructure. For example, loop-mediated isothermal amplification (LAMP)^14^ can return a diagnosis in just over 90 min (LamPORE device). Although faster than conventional RT-PCR, neither of these offer rapid results at a capacity that would facilitate mass screening or at a cost per device that would allow point-of-care testing in the home or in low-resource environments.^15,16^

Lateral-flow devices (LFD) are established tools for rapid diagnosis, giving results often in under 30 min and therefore can rapidly identify infected individuals. LFDs, such as the home-pregnancy test,^17^ use antibodies as detection units in both the stationary phase (test line bound to nitrocellulose) and as a coating for the mobile phase (on the surface of a gold nanoparticle). Upon binding the target analyte, the stationary and mobile phase form a “sandwich” with the analyte in the middle. The results are visible by the eye as a red or blue line depending on the precise gold formulation, although other nanomaterials, such as fluorescent particles, can be used.^18^ LFDs are typically cheap (compared to molecular methods), require little to no training or clinical infrastructure to use, and can be scaled up to enable large population testing. LFDs tend to have lower sensitivity (some false negatives) but high selectivity (few false positives). The cost-effectiveness and clinical usefulness of LFDs have been demonstrated by malaria rapid diagnostic tests,^19,20^ in the diagnosis of cutaneous leishmaniasis^21^ and in comparisons with more expensive RT-PCR approaches for Ebola diagnosis.^22^ Consequently, the appeal of LFDs in the COVID-19 pandemic is that their low cost and rapid turnaround time may enable mass testing of large populations.^23^ This could find asymptomatic individuals spreading the virus, who would not be identified by symptomatic RT-PCR testing only,^24−26^ currently the preferred option in most healthcare systems.

The first LFDs for the COVID-19 pandemic were designed to detect antibodies in patient blood samples produced in response to SARS-COV-2 infections.^27−29^ These were intended to report if a patient has previously been infected; not to indicate active infection, so could not effectively be used in screening/triage settings or mass testing for active infections. Antigen LFDs, in contrast, are designed to diagnose the presence of the virus i.e., an active infection. Several antigen lateral-flow tests, by late 2020, had passed Phase 3 testing in the United Kingdom,^30^ gained WHO “Emergency Use Listing” approval,^31^ or had emergency approval granted by The United States Food & Drug Administration.^32,33^ These devices all utilize antibodies as detection/capture units. To the best of our knowledge, these devices all use antibodies to target the nucleocapsid protein of SARS-COV-2. A university-based validation testing between LFDs and PCR confirmed that LFDs cannot detect lower viral loads but were estimated to be capable of identifying up to 85% of infections in the cohort trialed^26^ showing their potential for frequent, low-cost testing when deployed appropriately.

It is important to note that antibodies are not essential components in LFDs, and other recognition moieties could be used, including nucleic acids,^34^ glycans, and lectins.^35^ Glycan-based LFDs could offer advantages over other recognition moieties. For example, glycans are the site of pathogen adhesion during many viral infections^36,37^ especially respiratory viruses such as influenzas,^38^ and glycans can be chemically synthesized at scale. Glycan binding can also explore the “state” of a pathogen; for example, LecA/B are upregulated by *Pseudomonas aeruginosa* during infection.^39,40^ Furthermore, glycans are often more thermally robust than proteins^41^ making them ideal candidates for low-resource environments. Glycosylated gold nanoparticles (the mobile phase) are well established having been used in colorimetric/aggregation-based diagnostics, surface enhanced-Raman, and other bioassays.^42−45^ Despite this, glycans as capture units have not been widely applied in lateral flow^46^ and to the best of our knowledge have not been shown to function using clinical samples, only models.

We have previously reported that the S1 domain of the SARS-COV-2 spike protein can bind α,*N*-acetyl neuraminic acid (Neu5NAc), a sialic acid,^47^ and similar binding has been observed for other zoonotic coronaviruses toward sialic acids^48−50^ (e.g., MERS). The exact biological role of sialic acid binding is not yet understood for SARS-COV-2, with clear differences in its role in cell entry compared to MERS.^51^ Microarray, ELISA and STD NMR have been used to further demonstrate that sialic acids are receptors for the SARS-COV-2 spike protein.^52−54^ It has also emerged that sulfated glycosaminoglycans (including heparin sulfates) bind SARS-COV-2 spike protein, and can inhibit viral entry.^55−57^ Glycans (including those carrying terminal sialic acids) have been shown to participate in the angiotensin-converting enzyme 2 (ACE2) receptor binding during SARS-COV-2 cell adhesion/entry.^58^ Incorporation of α,*N*-acetyl neuraminic acid onto a polymer-stabilized glyconanoparticle platform enabled detection of (purified) spike protein in an LFD (5 μg·mL^–1^) and also detection of a pseudotyped lentivirus presenting the SARS-COV-2 spike protein at 1.5 × 10^4^ transduction units·mL^–1^ in a dipstick test.^47^

Herein, we demonstrate that glycan-based flow-through devices can detect SARS-COV-2 in heat-inactivated primary patient swabs and validate these initial results against RT-PCR. Compared to an LFD format, no test line was used, rather the sample is directly absorbed onto the nitrocellulose strip. Device optimization was achieved using a lentivirus and recombinant SARS-COV-2 spike protein showing that heat (or chemical) inactivation did not prevent usage. The prototype devices were then used with a panel of primary heat-inactivated swabs, demonstrating the principle that flow-through glyco-assays can be used to detect viral infection and hence that glyco-LFDs are feasible if suitable test lines can be developed. Furthermore, the devices were shown to detect recombinant mutant spike proteins showing that glycan-based detection may still detect variants of concern. This conceptual approach could be broadly applied to other pathogens/disease states and provide redundancy in testing regimes compared to using antibodies alone.

## Results

**Figure 1:**
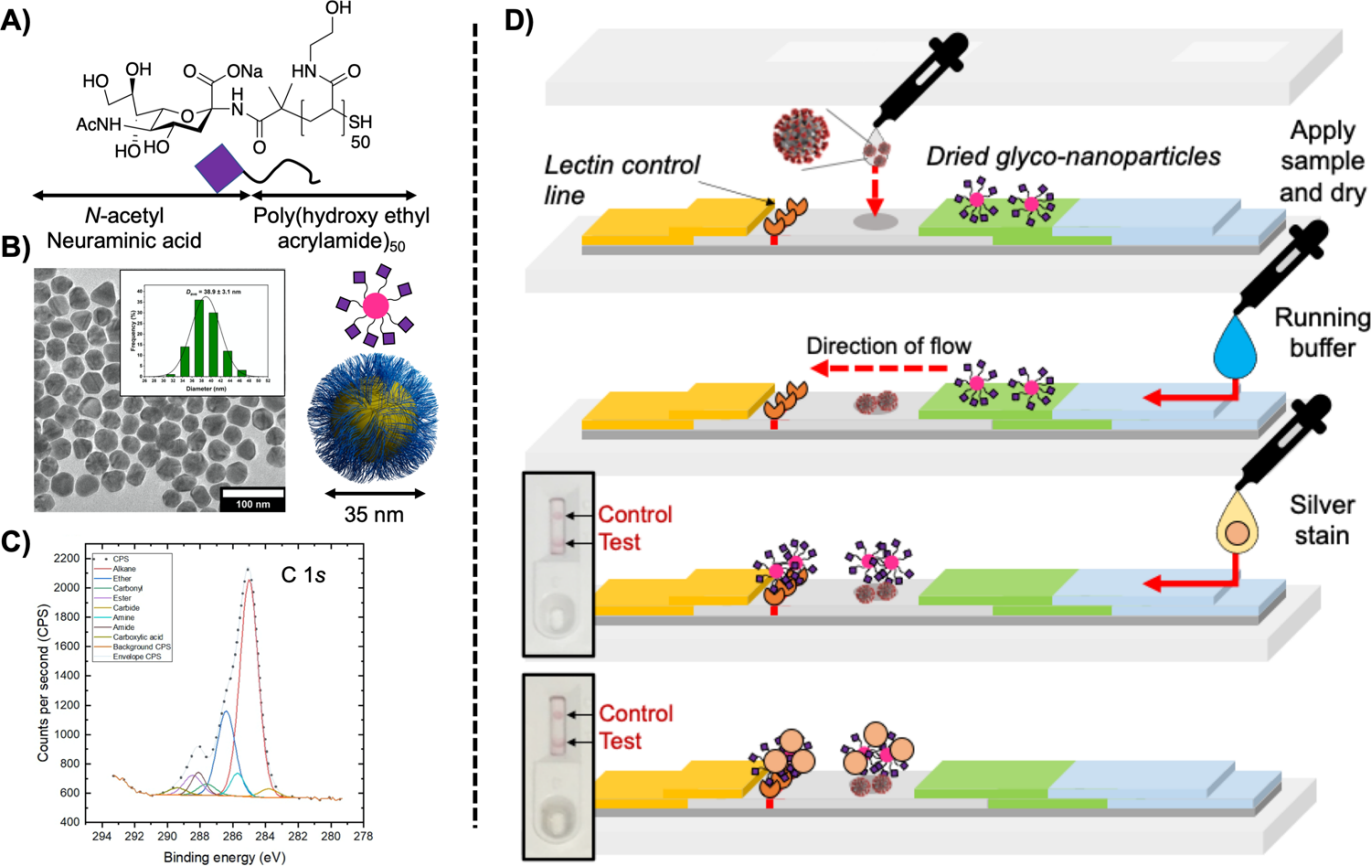
Nanoparticle synthesis and flow-through devices. (A) Neu5NAc-terminated polymer coating; (B) TEM micrograph of polymer-coated AuNPs; (C) C 1*s* portion of the XPS spectrum of polymer-coated AuNPs; and (D) flow-through device layout and assay procedure (top to bottom).

Our previously reported synthetic strategy to generate α-Neu5NAc-polymer-tethered gold nanoparticles was employed (Supporting Information).^47^ Telechelic poly(*N*-hydroxyethyl acrylamide), pHEA, was synthesized using RAFT (reversible addition–fragmentation chain transfer) polymerization, and 2-amino-2-deoxy-*N*-acetyl-neuraminic acid was conjugated to the ω-terminus by displacement of a pentafluorophenyl ester (allowing monitoring by ^19^F NMR).^59^ These polymers were then assembled onto gold nanoparticles (∼35 nm by TEM), Figure 1A–C, and characterized by DLS/UV–Vis (Supporting Information) and XPS (Figure 1C and Supporting Information). Just 10 mg of the glycan-terminated polymer can produce sufficient gold colloid for >2500 assays, highlighting the scalability of this approach. The use of a polymer linker between the particle and glycan provides colloidal stability and reduces nonspecific binding.

In a standard lateral-flow device, a test line is printed onto the paper to capture the antigen (e.g., a virus), which is then “sandwiched” by the nanoparticle detection unit. To streamline the development process, no test line was used, and instead, the patient sample is directly deposited and dried onto the strip with the viral components absorbing onto the stationary phase; hence, this is a flow-through, rather than lateral-flow, device.^23,60^ This removes the need for a validated, stable and specific test line, accelerating the development process and allowing us to prove the potential of glycan recognition for future complete lateral-flow devices. The setup of this approach is shown in Figure 1D, with the sample application, the flow of the glycan–gold conjugate, and then detection. Figure 1D also shows a silver-staining step, which can improve detection limits in flow-through devices (and LFDs) (discussed in detail later). The silver stain enhances the signal, as silver ions that are soluble in water are reduced to insoluble metallic silver catalyzed by the gold nanoparticles. This causes the silver to precipitate onto the surface of the gold increasing the signal.

**Figure 2:**
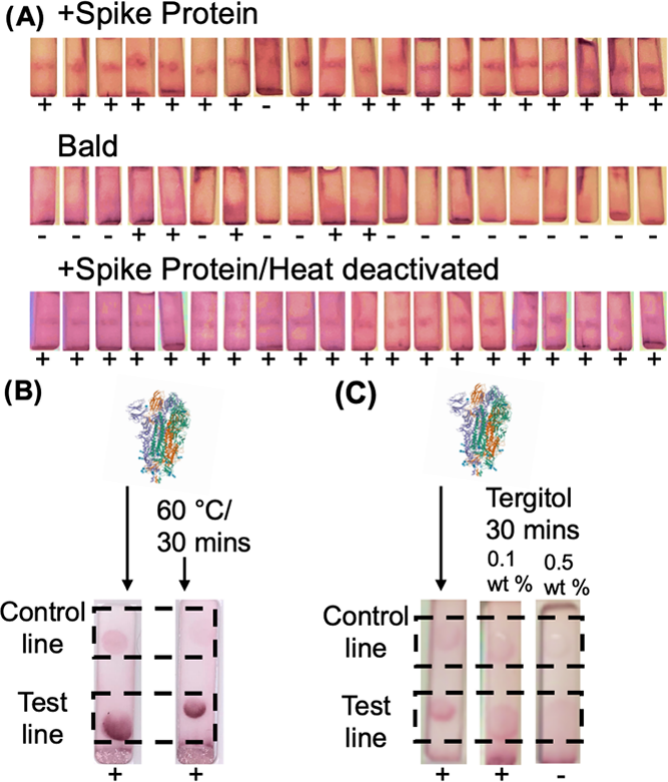
Device validation. (A) Photographs of the test line of lentivirus (no silver staining) positive for spike protein, negative (bald), and also after heat treatment at 60 °C for 30 min. The recombinant S1 domain of spike protein in flow-through devices; (B) heat treatment at 60 °C for 30 min [spike] = 0.25 mg·mL^–1^ (*Escherichia coli* expressed). (C) Tergitol treatment for 30 min [spike] = 0.5 mg·mL^–1^ (HEK293 expressed). Note control lines are not optimized but weak signals are present. “+” indicates a positive response and “–” indicates a negative response.

Flow-through cassettes were manufactured in-house, as described in the Supporting Information. SARS-COV-2 spike protein-bearing lentivirus was applied to the test line in 20 devices at 10^4^ transduction units·mL^–1^—a concentration within the expected viral range of COVID-positive patient respiratory swabs (Figure 2A).^61,62^ Nineteen out of 20 devices showed a positive result on the test line (no silver staining used). As a negative control, bald virus (without the spike protein) was also run in 20 cassettes. Five out of twenty showed potential weak positives, confirming the role of spike protein as the binding partner for the nanoparticles. The control line used in these devices was *Ricinus communis* agglutinin I (RCA_120_) lectin at 5 mg·mL^–1^, hence, a strong red line/crescent formed as the AuNPs were sequestered by the high concentration of RCA_120_ used. Later, the RCA_120_ control spot concentration was lowered to 1 mg·mL^–1^ to improve the performance. In the development of a “real” finished device, the control line also has to be validated, which is outside of the scope of this work. As 1 μL of the lentiviral solution was applied to each device, ∼10 transduction units/devices were applied, which would suggest a very low limit of detection. A possible explanation for this observation is that inert (nontransducible) particles, which also display spike protein, may contribute but are not counted in the transduction unit concentration, i.e., there are more potentially detectable particles than expected. Lentiviral vectors have been reported to show variance between the number of transduction units to genome copy in a range of 60–600, supporting this hypothesis.^63^

In current PCR testing laboratory protocols, nasal swabs are heat-treated during the processing cycle to sterilize and deactivate the virus prior to RNA-extraction steps.^64^ To evaluate if our flow-through device was compatible with a heat-inactivated virus, the lentivirus was heated to 60 °C for 30 min, and 20 repeats were run, and all cases gave a positive result. [Please note, when using primary swab samples, below, a different inactivation temperature is used, which was following a clinical workflow]. To probe the origin of thermal tolerance, a truncated SARS-COV-2 spike protein (expressed in-house in *E. coli*, see the Supporting Information) was heated to 60 °C for 30 min, then applied to devices (Figure 2B). As can be seen, heat treatment did not prevent binding. These observations show that glycan-based diagnostics may detect both intact and deactivated virus; this is also a condition of PCR, the current gold standard. The chemical deactivation medium was also explored to probe the tolerance. Tergitol NP-40 is a surfactant, which has been validated to inactive SARS-COV-2 at 0.1 and 0.5 wt % within 30 min.^65^Figure 2C shows devices with spike protein (expressed in HEK293 cells^47^) and Tergitol showing detection with 0.1 wt % but more spreading of the sample spot, which reduced the intensity. At the higher 0.5 wt %, the signal was reduced significantly due to the spreading of the test spot.

**Figure 3:**
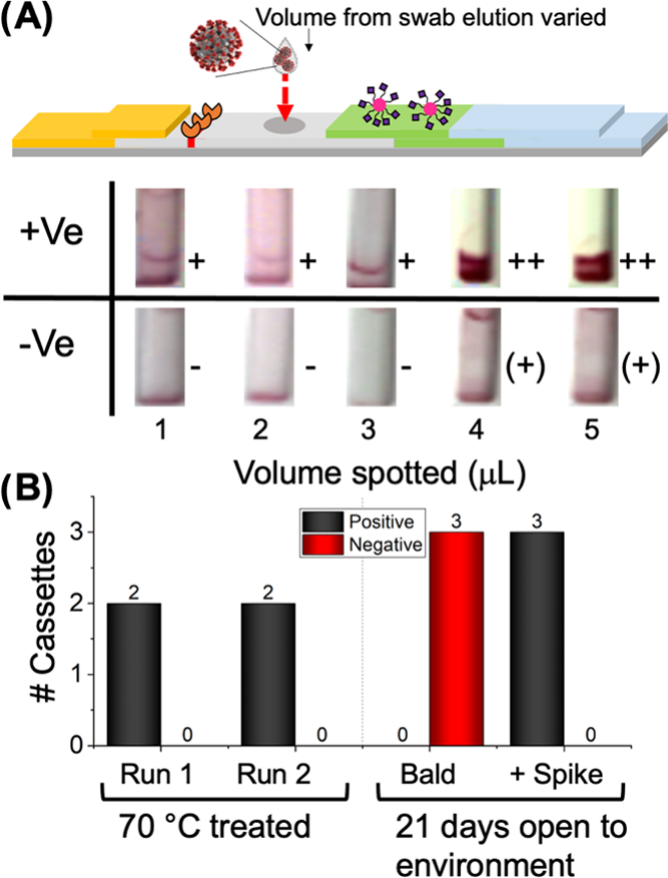
(A) Impact of sample volume applied to the test line. From 2 mL primary swab elution of Ct 8.3 (+ve) and a primary swab elution negative by RT-PCR (−ve), no silver staining used. (B) Impact of stress conditions on device function. Heat-treated devices tested with the swab sample (Ct 6.29) or after 21 days using indicated lentivirus (“Bald” ∼1 × 10^4^ LP·mL^–1^ and “+ Spike” 1.5 × 10^4^ TU·mL^–1^). “+” indicates a positive response on the test strips, “++” indicates a very strong positive response, “(+)” indicates a weak positive response, and “–” indicates a negative response.

As this flow-through approach requires direct addition of the swab extracts onto the test zone, the impact of volume applied was explored to optimize the deposition process. Figure 3A shows test zones of devices run as a function of the volume of a heat-inactivated primary nasal swab sample, which was validated as positive by RT-PCR (Ct = 8.3 from swab eluted with 2 mL of water) and an RT-PCR negative sample. Up to 3 μL (0.15 vol % of the total sample) could be applied to the test line without problems. However, further study (Table S8) highlighted problems with 3 μL with high viral load samples. Larger volumes (>3 μL) captured essentially all of the particles in flow, preventing the development of the control line. Some false positives also occurred with larger volumes, therefore 2 μL was chosen as the optimal application volume for experiments from here on.

Antibody-based LFDs (lateral-flow immunoassays) should not be exposed to extremes of humidity and heat, but it is expected that the glycan/polymer particles used here could be more robust. Devices were manufactured and left in the laboratory (on a shelf, with no desiccant) for 21 days, while some were baked in an oven for 12 h at 70 °C (Tables S5 and S9). Figure 3B shows the results of these preliminary stability tests, indicating that the tests retained function compared to cassettes not exposed to the atmosphere for 21 days (Tables S3 and S4) or subjected to heating (Table S9). It is important to note that the heat-treated devices did give weaker signals, but the conditions used for this were extreme and no silver staining was used at this point and hence the weaker signal. These initial robustness studies highlight the promise of glycopolymer systems; however, further studies and control line optimization are necessary. Device robustness is crucial for use “in the community” or in low-resource settings where cold chains are not established and more widely to reduce the number of failed devices.

**Figure 4:**
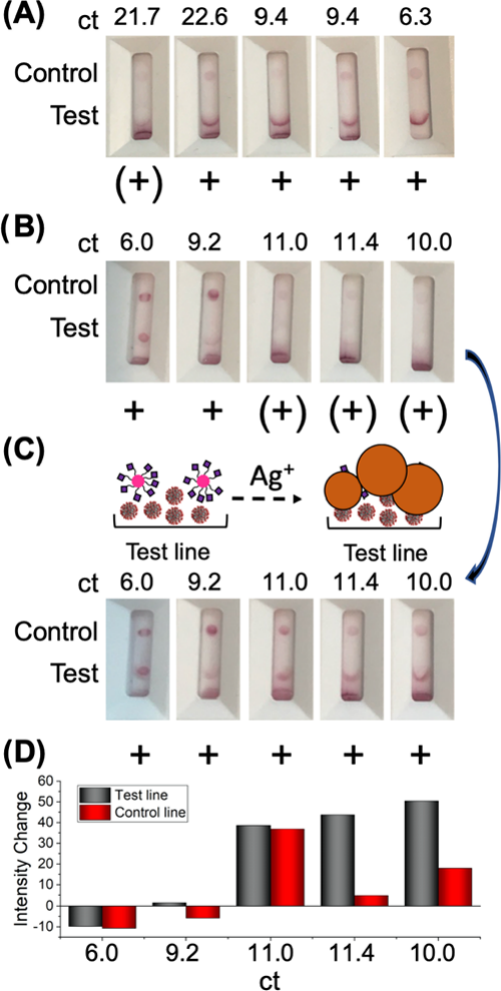
Flow-through device with clinical samples. Total 2 μL of the sample was applied to each test line. (A) Photographs taken after 20 min of buffer. (B) Photographs from different panels taken after 20 min of the buffer and then (C) subjected to silver-staining enhancement. (D) Impact of silver staining on signal intensity of control and test lines, obtained by image analysis.

Encouraged by the positive results with pseudotyped lentivirus, primary samples were the next step. For this, surplus nasal swabs eluates (which had been eluted and heat inactivated as part of clinical investigation of symptomatic patient/staff and assessed by RT-PCR) were used. These tests were not conducted blind, with the PCR result known to the user. After specimen application, devices were dried at 37 °C to ensure consistency across this study in terms of drying conditions. Figure 4A shows devices, following the addition of buffer: note that a lower Ct value indicates a higher viral load. A positive result (red line/spot at the test position) was clear, whereas control line/spot intensity varied between samples. It is crucial to note that a usable real-world device requires both control and test lines for a valid result. Converting Ct to viral concentration is not a linear relationship and varies between the methods used, but Figure 4 covers a wide range from weak to very strong positives. As these tests are “homemade”, there is likely to be more variance than in mass-manufactured devices and hence the silver-staining step employed here provides signal enhancement. Figure 4B exemplifies this with 5 other swab samples, which despite having relatively low Ct values gave weaker signals on the test line. After silver staining, Figure 4C shows that these (from Figure 4B) all now give clear and strong positives. Negative samples, after silver staining, did not lead to false positives (discussed further below in the context of larger sample numbers) (Tables S13–S15), unless longer developing times were used. Figure 4D shows the impact of silver staining on the signal intensity (from image analysis), confirming that low viral loads benefitted more from the signal increase, compared to higher viral loads (low Ct). It is notable that commercial lateral-flow diagnostics have a time window for reading results, as over-development can lead to false positives.

As previously discussed for the lentiviral data, as only 2 μL of the specimen is applied, the total number of viral particles/device is expected to be very low. A Ct of 26 (using RT-PCR) has been reported to give ∼100 PFU·mL^–1^ or ∼10^5^ RNA copies·mL^–1^.^66^ This would mean detection of ∼200 RNA copies per device or <1 PFU per device. Ct to PFU and RNA copy numbers are known to vary between RT-PCR machine, method, and calibration.^66−69^ Therefore, while Ct can give an indication of PFU and viral load, it is not an exact equivalence,^70^ and hence, for a test that detects the spike protein correlating these different measurements is challenging. Ct values used here were from the Abbott assay.^69,71^ It is important to note here that the numbers above do not include defective viral particles (e.g., capsid only and RNA-deficient particles),^72,73^ which may still have spike protein components (which is targeted in this work). In the case of the (cultured) Ebola virus, for example,^74^ depending on the passage number, the ratio of total viral particles to plaque-forming units (intact virus) has been reported in the range of 10^2^–10^5^, which, depending on the nature of particles, may contribute to diagnostic performance. To the best of our knowledge, the particle:PFU ratio is not available for SARS-COV-2, but we hypothesize that the detection limit may be enhanced due to these additional (non-plaque forming) viral particles or fragments of the released spike protein. Preliminary experiments on heat-treated purified SARS-COV-2 (from cell culture, not patients) showed higher limits of detection, supporting the hypothesis that defective particles may be contributing, rather than the release of spike protein from viral particles, which would also occur in this control.

**Figure 5:**
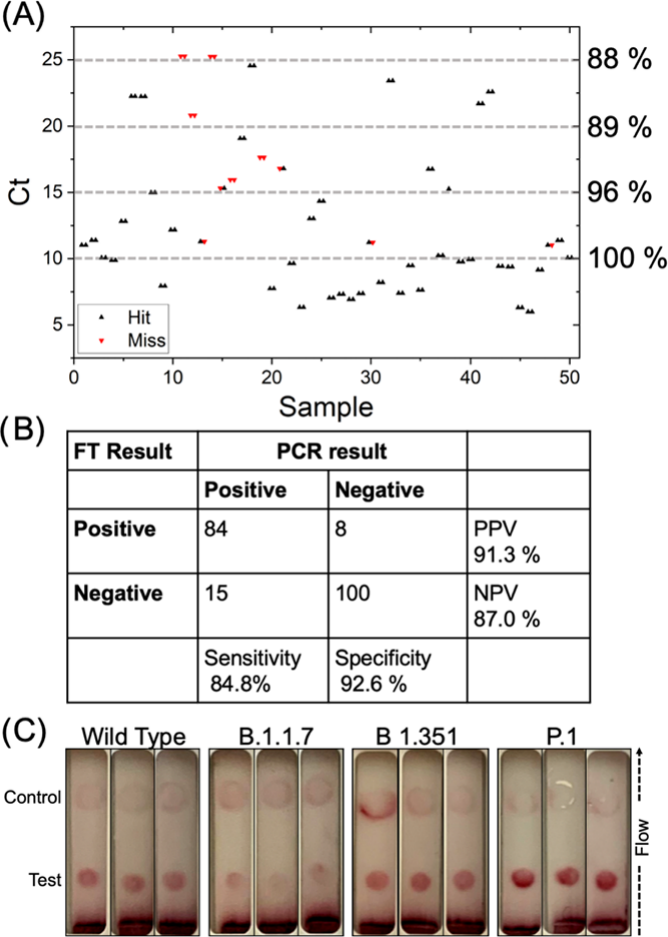
Flow-through (FT) device performance using heat-inactivated primary patient swabs after the silver-staining step (a positive result is test and control line being visible). (A) Results of device performance (hit or miss) as a function of Ct for devices ran alone. Thresholds indicated are the sensitivity as a function of the Ct value. (B) Confusion matrices after silver staining. Sensitivity = TP/(TP + FN); specificity = TN/(TN + FP); PPV = TP/(TP + FP); NPV = TN/(TN + FN). TP = true positive; TN = true negative; FN = false negative; and FP = false positive. (C) Devices using recombinant spike protein from variant strains. Sequence information in the Supporting Information. Larger versions of (A) can be found in Figure S16A,B for clarity.

Encouraged by the above results, a panel of 50 positive and 54 negative, PCR-validated patient-derived swab samples were tested (see the Supporting Information for how these were handled, including dry transport and heat inactivation.) Each sample was analyzed twice, on independent devices, treated in the analysis as an independent run, and reported as such in the results below. The tests were not run blind, and the Ct values were known to the user. Failed devices (where gold conjugate did not flow, for example) were excluded from the analysis (1 positive sample device and 2 negative sample devices). As above, all samples were dried onto the devices at 37 °C before running to ensure consistency. Figure 5A shows the distribution of positive samples as a function of the Ct values after silver staining, with high viral loads (lower Ct) giving fewer false negatives, as would be expected. Figure 5A is annotated showing the sensitivity (% true positives) by the Ct value. Analysis of non-silver-stained devices is provided in the Supporting Information for comparison purposes.

Confusion matrices were produced from both positive and negative sample sets (Figure 5B). After silver staining, a sensitivity value of 85% and specificity of 93% were achieved. This sensitivity is comparable to some commercial LFDs,^66^ whereas the specificity is lower. Before silver staining (where control lines were not always visible and hence judged by the test line presence only), a lower sensitivity (68%) but higher specificity (96%) was observed. The total number of false positives was 8 (from 6 samples) across the study. Considering that this is a prototype, the values are promising. To the best of our knowledge, this is the first example of a flow-through glyco-assay assessed with clinical samples, providing proof of principle that glycan binding could be exploited in a complete lateral-flow type device to complement antibody-based systems.

SARS-COV-2 variants with mutations in the spike protein have (and continue to) emerged, and any diagnostics should retain the ability to detect these. Davis and co-workers have reported that the B1.1.7 and B1.351 spike mutants have reduced NMR STD signal (i.e., weaker contacts) to the NAc protons of α2,3-α sialyllactoside compared to the wild-type, and hence there is potential that the glycan-binding affinity may be decreased.^52^ To test the impact of this, 3 mutant truncated spike proteins, B1.1.7, B.1.351, and P1 (variants first detected in Kent (U.K.), South Africa, and Brazil), were expressed in *E. coli* and tested in our devices. In all cases, a positive test line was seen (Figure 5C, no silver staining), showing that detection capability is retained. It is crucial to again note that binding affinity does not relate linearly to signal output in flow-through (or lateral-flow) devices and hence this does not rule out differences in the individual protein/glycan interactions.

Influenza has haemagglutinins and neuraminidases, which target sialosides (including *N*-acetyl neuraminic acid),^75^ and sialic acid nanoparticles, which bind influenza, are well known,^76,77^ so it was important to consider cross-reactivity. Heating is known to reduce haemagglutination activity;^78^ hence, our specificity (above) might have been improved by the heat inactivation of the sample. To explore influenza cross-reactivity, haemagglutinins from H1N1, H3N2, H7N8, and H7N3 as well as betapropiolactone (BPL)-inactivated influenza virions were tested and the devices are shown in the Supporting Information. H3N2 haemagglutinins were detected in the devices but H1N1, H7N9, and H7N3 haemagglutinins were not, noting relatively high concentrations were used (0.5 mg·mL^–1^). In contrast, using intact influenza virus there was no cross-reactivity observed. A further control of heat-inactivated SARS-COV-2 remained detectable under these conditions (Supporting Information). The lack of apparent influenza cross-reactivity can be attributed to the effective low haemagglutinin concentration on the viral surface, compared to using just “pure” protein along with differential absorption onto the nitrocellulose. From a structural biology perspective, haemagglutinins make binding contacts to not only the terminal glycan used here (Neu5NAc) but can also contact linker units (e.g the lactose, in sialylactose). Our preliminary data^47^ and additional thermal shift assays (Supporting Information) suggest that the SARS-COV-2 spike protein had a similar affinity toward Neu5NAc as to sialyllactose (2.3 and 2.6). Hence, the use of the Neu5NAc monosaccharide as the detection unit may lead to reduced overall affinity toward influenzas, while maintaining SARS-COV-2 affinity, and hence providing some selectivity in the flow-through format.

## Discussion

Here, we have demonstrated a prototype flow-through device, which is capable of detecting SARS-COV-2 by exploiting the interaction between α-*N*-acetyl neuraminic acid and the viral spike protein. Rather than a traditional lateral-flow design where there is a capture unit on the stationary phase (“test line”), we developed our system so that the primary sample (in this case derived from nasal swabs) was directly deposited as the test line and hence is a “flow-through” device. This approach removed the need to develop a test line, speeding up the initial development process and allowing us to prove the principle that glycans could be used in complete lateral-flow devices with primary samples. Crucial to achieving this is the use of a polymeric coating, which reduces nonspecific interaction with any deposited biological components (e.g mucus, cell debris) as well as providing the tether to display the glycan. Using a lentiviral model, the flow-through devices were specific toward spike-bearing lentiviruses, compared to bald lentiviruses. Using recombinant, truncated, spike protein, we demonstrated that the protein retains sialic acid-binding capacity even after heating or limited detergent treatment. This observation shows that this device may detect damaged viruses and hence cannot be claimed to only detect intact viruses (similar to other diagnostic tools for SARS-COV-2). Using a panel of RT-PCR-validated swab samples, these prototype flow devices were shown to achieve, after silver staining, 85% sensitivity and 93% specificity, using Ct values as high as 25. The apparently low detection limit may be in part due to the detection of defective viral particles, which also bear the spike protein. This will require further studies to validate their contribution and the role of using heat-inactivated swabs and their handling chain. Further optimization of the device and running buffers are expected to lead to improvements, especially to further reduce any nonspecific interactions, as well as the potential to develop a full lateral-flow device.

With any diagnostic or sensor, there is potential for cross-interaction with other agents. Cross-reactivity with two influenza strains (H1N1 and H3N2) was not seen, even though the nanoparticles do have an affinity toward H3N2 haemagglutinins, which may be due to differential absorbance to the test zone or differences in overall detection limits. The molecular details of recently reported spike protein mutations (including the H69/V70 deletions) on the actual binding affinity toward sialosides (and hence this detection method) are still under study.^79^ The devices developed here were shown to be capable of detecting recombinant spike proteins from several variants, demonstrating that these mutations do not remove glycan-binding function. Future work will further explore the roles of sample preparation including the heat-inactivation step, mechanism of application of specimens, and fundamental studies of the glycan recognition function and its biochemical basis. Consideration must also be given to removing the need for a pipette as an application system to the device, followed by the time delay for drying the specimen onto the strip. Both of these could be improved, or more ideally developed into a complete lateral-flow (test line) device, which will be explored in the future. The evidence provided here shows that glycan flow technology (lateral-flow and flow-through glyco-assays) could be translated to clinical settings to be used alongside more traditional antibody-based approaches.

## Materials and Methods

Please see the Supporting Information for complete experimental procedures.

## Supporting Information Available

Complete chemical synthesis/characterization and biological methods; device manufacture; uncropped and unedited images of all flow-through devices used in this work; and additional control experiments (PDF)

## Notes

This study used remnant elutions from nasal swab samples routinely collected from symptomatic staff/patients at the University Hospital Coventry and Warwickshire NHS Trust and tested by standard PCR protocols employing the Abbott RealTime SARS-COV-2 assay (09N77-095) during April–September 2020. As this was an evaluation study using the left-over anonymized material (which had been heat-treated to render acellular), no written informed consent was obtained, although the project was registered with the local COVID-19 research committee.
