# Supplementary material for: Glycan-Based Flow-Through Device for the Detection of SARS-COV-2
Source: ACS Sens. 2021 Oct 11;6(10):3696–705. doi: 10.1021/acssensors.1c01470 (PMC8525701; doi:10.1021/acssensors.1c01470)
Supplement: Supplementary file 1 — se1c01470_si_001.pdf [file se1c01470_si_001.pdf]

Supporting Information for

Glycan-Based Flow-Through Device for the Detection of SARS-COV-2

Alexander N. Baker,[a,‡] Sarah-Jane Richards,[a,‡] Sarojini Pandey,[e] Collette S. Guy,[a,b] Ashfaq Ahmad,[a,c] Muhammad Hasan,[a,c] Caroline I. Biggs,[a] Panagiotis G. Georgiou,[a] Alexander J. Zwetsloot,[c] Anne Straube,[c] Simone Dedola,[f] Robert A. Field,[g] Neil R Anderson,[e] Marc Walker,[d] Dimitris Grammatopoulos,[c,e] and Matthew I. Gibson [a,c] *

a) Department of Chemistry, University of Warwick, UK, CV4 7AL

b) School of Life Sciences, University of Warwick, UK, CV4 7AL

c) Warwick Medical School, University of Warwick, UK, CV4 7AL

d) Department of Physics, University of Warwick, UK, CV4 7AL

e) Institute of Precision Diagnostics and Translational Medicine, University Hospitals Coventry and Warwickshire NHS Trust, Clifford Bridge Road, Coventry, CV2 2DX

f) Iceni Diagnostics Ltd, Norwich Research Park, Norwich, NR4 7GJ

g) Department of Chemistry and Manchester Institute of Biotechnology, University of Manchester, Manchester, UK, M1 7DN

Corresponding author contact details. M.i.gibson@warwick.ac.uk

Contents

Contents 3

Physical and Analytical Methods 6

NMR Spectroscopy 6

Size Exclusion Chromatography 6

X-ray Photoelectron Spectroscopy (XPS) 6

Dynamic Light Scattering 7

UV-vis Spectroscopy 7

Transmission Electron Microscopy 8

Protein Thermal Shift Assay 8

Materials 9

Swab Samples 10

Synthetic Methods 12

Polymerization of 2-hydroxyethyl acrylamide (DP50) 12

DP50 Poly(N-hydroxyethyl acrylamide) glycan functionalisation using 2-amino-2-deoxy-N-acetyl-D-neuraminic acid 14

Citrate-stabilised 35 nm Gold Nanoparticle Synthesis 16

Gold Nanoparticle Polymer Coating Functionalisation 16

Characterization of 2-Azido-2-deoxy-N-acetyl-D-neuraminic acid 22

Recombinant Expression and Purification of truncated SARS-COV-2 Spike (S1) Protein (first 300 amino acids) in E.coli 24

SARS-COV-2 spike protein variants 26

Expression and purification of SARS-COV-2 Spike (S1) in HEK293 Cells 27

Flow-Through Cassette Production, Running and Analysis Protocols...................................29

Flow-Through Cassette Production, Running and Analysis Protocols...............................29

Protocol for Manufacturing Flow-Through Cassettes 29

Protocol for Conjugate Pad Production 30

10× Conjugate Pad Buffer 30

Control Line Addition...31

Sample Line Addition 31

Protocol for running flow-through tests..31

Silver Staining Procedure 31

Flow-through assay buffer - 10× HEPES buffer (20% PVP_{400}) in 100 mL H_2O 31

Protocol for analysing flow-through tests to determine signal intensity and intensity change 32

Additional Data and Figures 33

Flow-Through Strips and Plotted Data 33

Cassettes from Pseudotyped Lentivirus Experiments..34

Cassettes from Swab Sample Experiments Not Used to Determine Specificity and Selectivity 38

Cassettes from Swab Sample Experiments Used to Determine Specificity and Selectivity42

Analysis of Patient Samples Before Silver Staining...50

Analysis of Patient Samples – Larger Versions of Figure 5A and Figure S14....................51

Cassettes from SARS-COV-2 Spike Protein S1, SARS-COV-2 heat-inactivated virus, Influenza viruses and Hemagglutinin Experiments 55

Spike (S1) Protein Thermal Shift Binding Analysis 61

References 62

Physical and Analytical Methods

NMR Spectroscopy

^{1}H-NMR, ^{13}C-NMR and ^{19}F-NMR spectra were recorded at 300 MHz or 400 MHz on a Bruker DPX-300 or DPX-400 spectrometer respectively, with deuterium oxide (D_2O) as the solvent. Chemical shifts of protons are reported as δ in parts per million (ppm) and are relative to D_2O (4.79).

Size Exclusion Chromatography

Size exclusion chromatography (SEC) analysis was performed on an Agilent Infinity II MDS instrument equipped with differential refractive index (DRI), viscometry (VS), dual angle light scatter (LS) and variable wavelength UV detectors. The system was equipped with 2 x PLgel mixed D columns (300 x 7.5 mm) and a PLgel 5 µm guard column. The mobile phase used was DMF (HPLC grade) containing 5 mM NH_4BF_4 at 50 °C at flow rate of 1.0 mL.min^{-1}. Poly(methyl methacrylate) (PMMA) standards (Agilent EasyVials) were used for calibration between 955,000 – 550 g.mol^{-1}. Analyte samples were filtered through a nylon membrane with 0.22 μm pore size before injection. Number average molecular weights (M_n), weight average molecular weights (M_w) and dispersities ($Đ_M$ = M_w/M_n) were determined by conventional calibration using Agilent GPC/SEC software.

X-ray Photoelectron Spectroscopy (XPS)

The samples were attached to electrically-conductive carbon tape, mounted on to a sample bar and loaded in to a Kratos Axis Ultra DLD spectrometer which possesses a base pressure below 1 x 10^{-10} mbar. XPS measurements were performed in the main analysis chamber, with the sample being illuminated using a monochromated Al Kα x-ray source. The measurements were conducted at room temperature and at a take-off angle of 90° with respect to the surface

parallel. The core level spectra were recorded using a pass energy of 20 eV (resolution approx. 0.4 eV), from an analysis area of 300 μm x 700 μm. The spectrometer work function and binding energy scale of the spectrometer were calibrated using the Fermi edge and $3d_{5/2}$ peak recorded from a polycrystalline Ag sample prior to the commencement of the experiments. In order to prevent surface charging the surface was flooded with a beam of low energy electrons throughout the experiment and this necessitated recalibration of the binding energy scale. To achieve this, the C-C/C-H component of the C 1*s* spectrum was referenced to 285.0 eV. The data were analysed in the CasaXPS package, using Shirley backgrounds and mixed Gaussian-Lorentzian (Voigt) lineshapes. For compositional analysis, the analyser transmission function has been determined using clean metallic foils to determine the detection efficiency across the full binding energy range.

Dynamic Light Scattering

Hydrodynamic diameters (Dh) and size distributions of particles were determined by dynamic light scattering (DLS) using a Malvern Zetasizer Nano ZS with a 4 mW He-Ne 633 nm laser module operating at 25 °C. Measurements were carried out at an angle of 173° (back scattering), and results were analysed using Malvern DTS 7.03 software. All determinations were repeated 5 times with at least 10 measurements recorded for each run. D_h values were calculated using the Stokes-Einstein equation where particles are assumed to be spherical.

UV-vis Spectroscopy

Absorbance measurements were recorded on an Agilent Cary 60 UV-Vis Spectrophotometer and on a BioTek Epoch microplate reader.

Transmission Electron Microscopy

Dry-state stained TEM imaging was performed on a JEOL JEM-2100Plus microscope operating at an acceleration voltage of 200 kV. All dry-state samples were diluted with deionized water and then deposited onto formvar-coated copper grids.

Flow-through Cassette Image Collection

Images of the flow-through cassettes were collected on an iPhone XR or an iPhone 7, using standard/automatic photo settings i.e. no manual adjustments were made by the authors to improve photo quality on capture. Images in main paper have been cropped and brightness/contrast changed. Original images are included in full in this supporting information.

Protein Thermal Shift Assay

The thermal shift reaction was performed with a BioRad CFX96 real-time PCR machine. The sample was heated from 25 °C to 95 °C and the fluorescence intensity change monitored using the Protein Thermal Shift™ Dye kit (Thermo Fisher Scientific, Cat # 4461146). Analysis for binding induced shifts in thermal transition was performed in PBS buffer with Precision Melt Analysis Software provided by the manufacturer (BioRad) and a protein concentration of 0.2 mg/mL. The data was collected over 5 runs for each sugar and sugar concentration. Glucose and Galactose data are from a prior publication,[1] with the addition of sialyllactoses here.

Materials

All chemicals were used as supplied unless otherwise stated. *N*-Hydroxyethyl acrylamide (97 %), 4,4'-azobis(4-cyanovaleric acid) (98 %), triethylamine (> 99%), sodium citrate tribasic dihydrate (> 99 %), gold(III) chloride trihydrate (99.9%), potassium phosphate tri basic (≥ 98%, reagent grade), deuterium oxide (D_2O, 99.9%), diethyl ether ((≥ 99.8%, ACS reagent grade), sodium azide (≥ 99.5%, reagent plus grade), methanol (≥ 99.8%, ACS reagent grade), toluene (≥ 99.7%,), Tween-20 (molecular biology grade), HEPES, PVP40 (poly(vinyl pyrrolidone)$_{400}$ (Average Mw ~40,000)), sucrose (Bioultra grade), acetone (≥ 99%) and a silver staining kit (Silver Enhancer Kit), were purchased from Sigma-Aldrich. Anhydrous trehalose was purchased from Alfa Aesar. DMF (>99%) was purchased from Acros Organics. Sodium chloride (≥ 99.5%) and calcium chloride were purchased from Thermo Fisher Scientific.

Nitrocellulose Immunopore RP 90-150 s/4cm 25mm was purchased from GE Healthcare. Lateral flow backing cards 60mm by 301.58mm (KN-PS1060.45 with KN211 adhesive) and lateral flow cassettes (KN-CT105) were purchased from Kenosha Tapes. Cellulose fibre wick material 20 cm by 30 cm by 0.825 mm (290 gsm and 180 mL/min) (Surewick CFSP223000) was purchased from EMD Millipore. Glass fibre conjugate pads (GFCP103000) 10 mm by 300 mm was purchased from Merck. Thick chromatography paper (for sample pads), Grade 237, Ahlstrom 20 cm by 20 cm were purchased from VWR International.

Ricinus communis Agglutinin I (RCA_{120}) was purchased from Vector Laboratories.

Spike (SARS-COV2) pseudotyped lentivirus (*Luc* Reporter) (Catalogue number: 79942, Lot number: 200730) and Bald lentiviral pseudovirion (*Luc* reporter) (Catalogue number: 79943, Lot number: 200727) were purchased from amsbio.

Influenza A virus (A/Brisbane/10/2007 (H3N2)) BPL-inactivated (Catalogue number: NR-19321), Influenza A virus (A/Puerto Rico/8/1934 (H1N1)) BPL-inactivated (Catalogue

number: NR-19325), H7 Hemagglutinin (HA) protein from influenza virus (A/Canada/rv444/2004 (H7N3)) – recombinant from Baculovirus (Catalogue number: NR-43740), H7 Hemagglutinin (HA) protein from influenza virus (A/Shanghai/1/2013 (H7N9)) – recombinant from Baculovirus (Catalogue number: NR-44079), H3 Hemagglutinin (HA) protein from influenza virus (A/New York/55/2004 (H3N2)) – recombinant from Baculovirus (Catalogue number: NR-19241), H1 Hemagglutinin (HA) protein with C-terminal histidine tag from influenza virus (A/Brisbane/59/2007 (H1N1)) – recombinant from Baculovirus (Catalogue number: NR-28607) and SARS-related Coronavirus 2 (SARS-COV-2) (USA-WA1/2020) heat-inactivated at 65°C for 30 minutes (Catalogue number: NR-52286) were obtained through BEI Resources.

Water used for buffers was MilliQ grade >18.2 mΩ resistance.

Swab Samples

This study used remnant elutions from nasal, or nasal + oral swab samples collected from symptomatic staff/patients at the University Hospital Coventry and Warwickshire NHS Trust and routinely tested by standard PCR protocols employing the Abbott assay (Ref: 09N77-095, https://www.molecular.abbott/sal/9N77-095_SARS-CoV-2_US_EUA_Amp_PI.pdf) during April-September 2020.[2] As this evaluation study used left-over anonymized material no written informed consent was obtained, although the project was registered with the local COVID-19 research committee.

Dry cotton swabs, one nose and one throat, were obtained in a single universal container for transport. To each primary swab sample was added 2000 µL of molecular grade water (if one swab) or 2500 µL of molecular grade water (if two swabs) in universal container. These were then vortexed and allowed to settle for 5 minutes. All liquid was transferred from primary container into 13 mm × 75 mm tube. These tubes were heat inactivated at 85 °C for 10 minutes.

All testing was conducted on samples which had not been frozen, but had been stored in a fridge, and tested within 48 hrs of receipt.

Synthetic Methods

The following synthetic methods have been previously reported,[1] but have been provided for the reader as the glycopolymer synthesis and AuNP functionalisation have been optimised and materials were made specifically for this work. Note the synthesis of 2-azido-2-deoxy-*N*-acetyl-D-neuraminic acid and the SARS-COV2,S1 Spike protein can be found in the previous report[1] but a full characterisation of the 2-azido-2-deoxy-*N*-acetyl-D-neuraminic acid has been provided as this was synthesised again here.

Polymerization of 2-hydroxyethyl acrylamide (DP50)

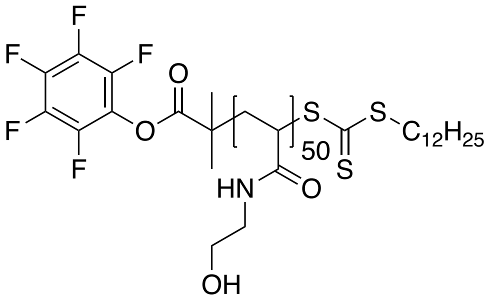

2.7635 g (24.00 mmol) of 2-hydroxyethyl acrylamide, 0.0607 g (0.22 mmol) of ACVA and 0.5273 g (2.87 mmol) of PFP-DMP (synthesised previously[1]) was added to 22 mL 1:1 toluene:methanol and degassed with nitrogen for 30 minutes. The reaction vessel was stirred and heated at 70 °C for 2 hours. The solvent was removed under vacuum. The crude product was dissolved in the minimum amount of methanol. Diethyl ether cooled in liquid nitrogen was added to the methanol to form a precipitate. The mixture was centrifuged for 2 minutes at 13 krpm and the liquid decanted off. The solid was dissolved in methanol and removed under vacuum to give a yellow crystalline solid. δ_H (300 MHz, D_2O) 8.31 - 7.97 (23H, m, N*H*), 3.99 - 3.55 (86H, m, NHCH_2), 3.55 - 3.09 (100H, m, CH_2OH & SCH_2), 2.49 - 1.90 (46H, m, CH_2C*H*C(O) & C(CH_3)$_2$), 1.90 - 0.98 (110H, m, CH_2CHC(O) & CH_2CH_2CH_2CH_2CH_2CH_2CH_2CH_2C$H_2$$CH_2CH_3$), 0.84 - 0.72 (5H, m, C$H_2CH_3$).

$M_{n,Theoretical}$ = 3400 g.mol^{-1}. $M_{n,NMR}$ = 5500 g.mol^{-1}. SEC (5 mM NH_4BF_4 in DMF) $M_{n, SEC\ RI}$ = 6400 g.mol^{-1} ($DP_{PHEA, SEC}$ = 50), $Đ_{M, SEC\ RI}$ = 1.27. Yield - 75%.

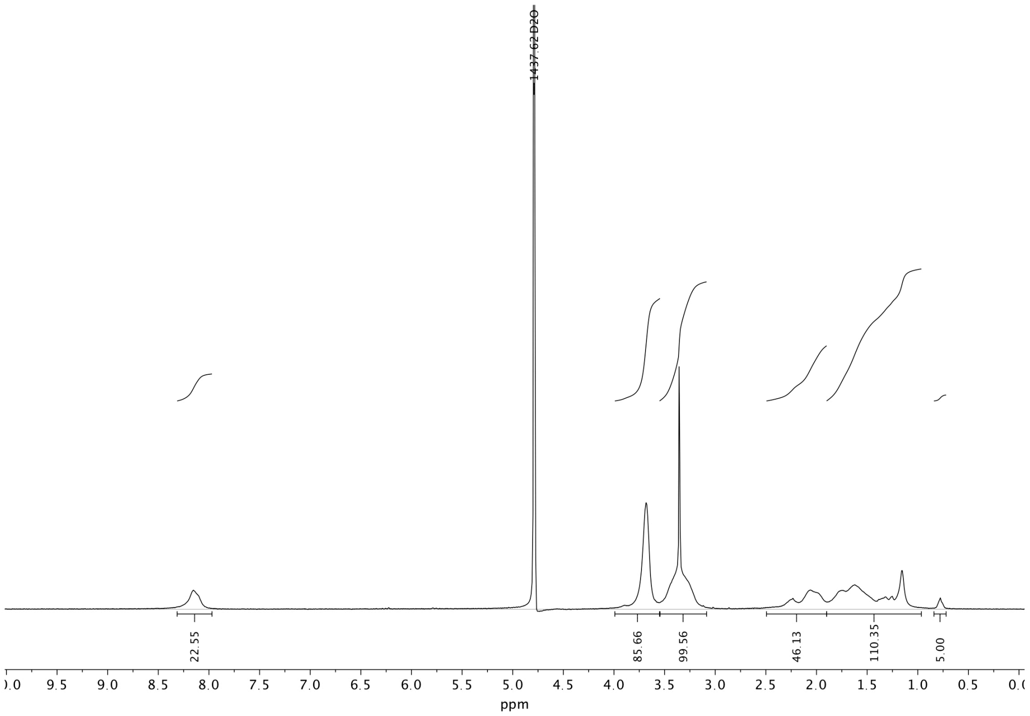

Figure S1. ^{1}H NMR spectrum of DP50 PHEA

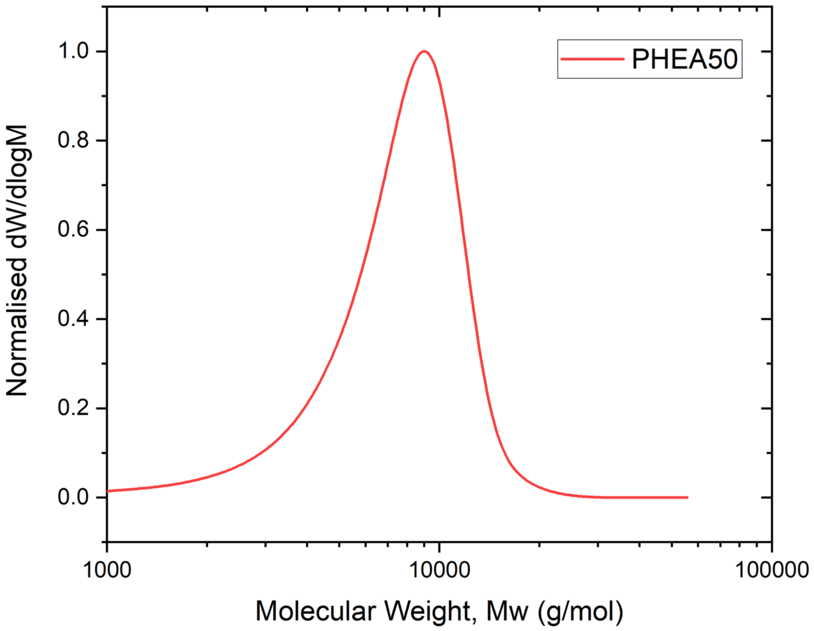

Figure S2. Normalised size exclusion chromatography RI molecular weight distribution of telechelic PHEA50 obtained in DMF versus PMMA standards.

DP50 Poly(N-hydroxyethyl acrylamide) glycan functionalisation using 2-amino-2-deoxy-N-acetyl-D-neuraminic acid

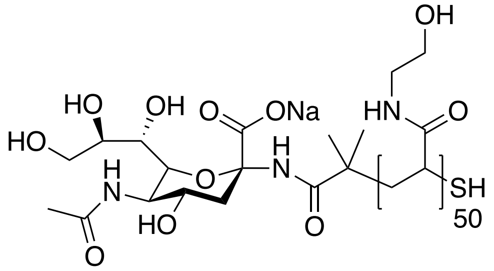

0.1 g (0.016 mmol) of poly(2-hydroxyethyl acrylamide)$_{50}$ and 25 mg (0.64 mmol) of 2-amino-2-deoxy-*N*-acetyl-D-neuraminic acid were added to 3 mL of DMF containing 100 μL TEA. The reaction was stirred at RTP for 16 hours. Solvent was removed under vacuum. The crude product was dissolved in the minimum amount of methanol. Diethyl ether cooled in liquid nitrogen was added to the methanol to form a precipitate. The mixture was centrifuged for 2 minutes at 13 krpm and the liquid decanted off. The solid was dissolved in methanol and solvent removed under vacuum to give an orange/brown crystalline solid. Loss of fluorine signals in the ^{19}F NMR was used to indicate the reaction had gone to completion. δ_H (400 MHz, D_2O) 3.99 - 3.55 (~117H, m, NHCH_2 & glycan protons), 3.55 - 3.09 (~98H, m, CH_2OH & SCH_2 & glycan protons), 2.49 - 1.90 (~63H, m, CH_2C*H*C(O), C(CH_3)$_2$ & glycan protons), 1.90 - 0.98 (~95H, m, CH_2CHC(O) & glycan protons).

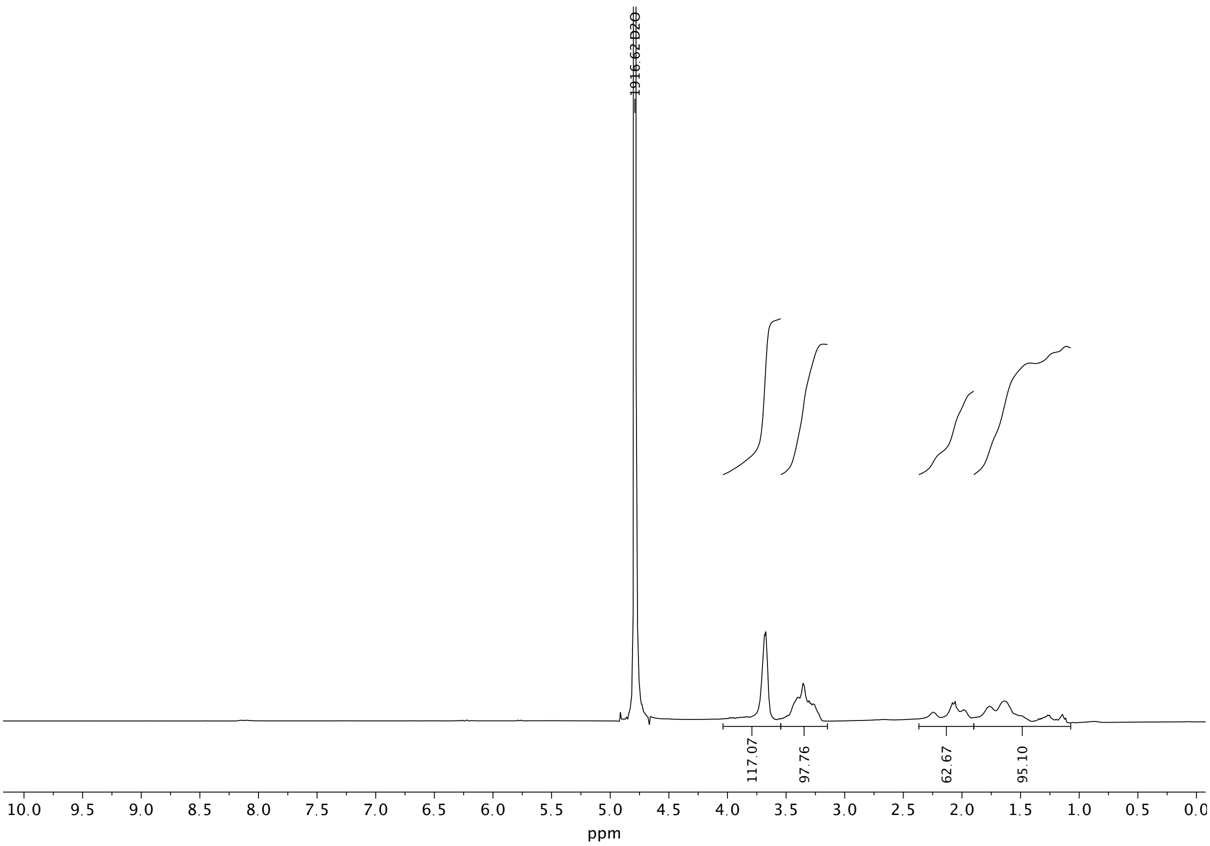

Figure S3. ^{1}H NMR of 2-amino-2-deoxy-*N*-acetyl-D-neuraminic acid functionalised poly(*N*-hydroxyethyl acrylamide)$_{50}$.

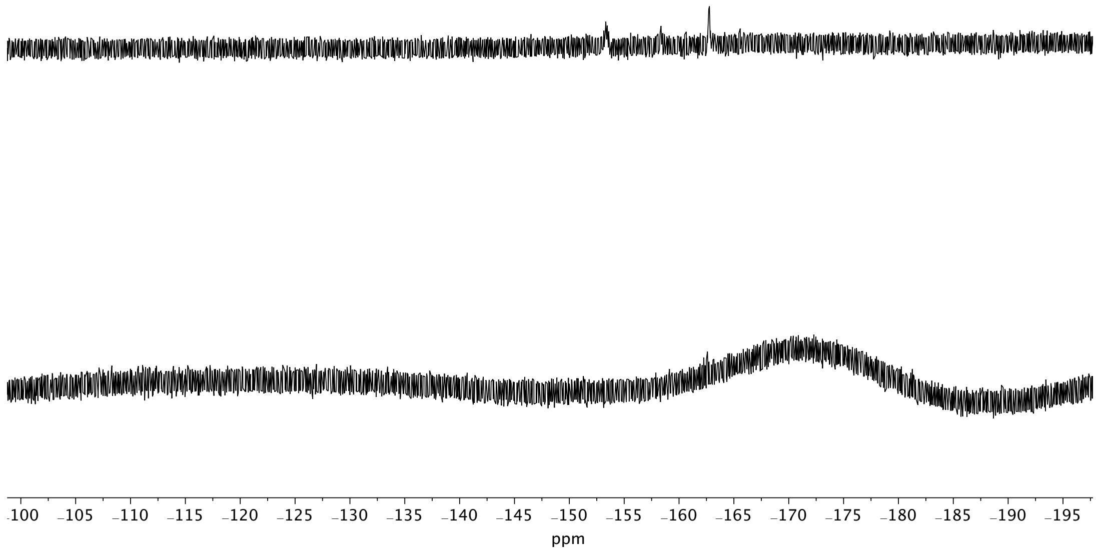

Figure S4. ^{19}F NMR before (Top) and after (Bottom) reaction with α2-amino-2-deoxy-*N*-acetyl-D-neuraminic acid functionalization.

Citrate-stabilised 35 nm Gold Nanoparticle Synthesis

35 nm gold nanoparticles were synthesised by a modified step growth method developed by Bastús *et al.*[3] A solution of 2.2 mM sodium citrate in Milli-Q water (150 mL) was heated under reflux for 15 min while vigorously stirring. After boiling had commenced, 1 mL of $HAuCl_4$ (25 mM) was injected. The colour of the solution changed from yellow to bluish gray and then to soft pink in 10 min, 1 mL was taken for DLS and UV/Vis analysis. Immediately after the synthesis of the Au seeds and in the same reaction vessel, the reaction was cooled until the temperature of the solution reached 90 °C. Then, 1 mL of a $HAuCl_4$ solution (25 mM) was injected. After 20 min, the reaction was finished. This process was repeated twice. After that, the sample was diluted by adding 85 mL of MilliQ water and 3.1 mL of 60 mM sodium citrate. This solution was then used as a seed solution, and three further portions of 1.6 mL of 25 mM $HAuCl_4$ were added with 20 min between each addition. Following completion of this step, 1 mL was taken for DLS and UV/Vis analysis. The sample was diluted by adding 135 mL of MilliQ water and 4.9 mL of 60 mM sodium citrate. This solution was then used as a seed solution, and the process was repeated with three further additions of 2.5 mL of 25 mM $HAuCl_4$, this solution was analysed by DLS and UV/Vis. When the target size of 35 nm was reached, the solution was cooled, and a sample taken for TEM analysis.

Gold Nanoparticle Polymer Coating Functionalisation

1 mg of glycopolymer was agitated overnight with 10 mL of 35 nm AuNPs ~2 Abs at UV_{max}. The solution was centrifuged at 8 krpm for 30 minutes and the pellet resuspended in 10 mL of water, the solution was centrifuged again at 8 krpm for 30 minutes and the pellet resuspended in 1 mL aliquots and centrifuged at 8 krpm for 10 minutes. The pellets were combined into a 1 mL solution with an absorbance at UV_{max} of ~10 Abs.

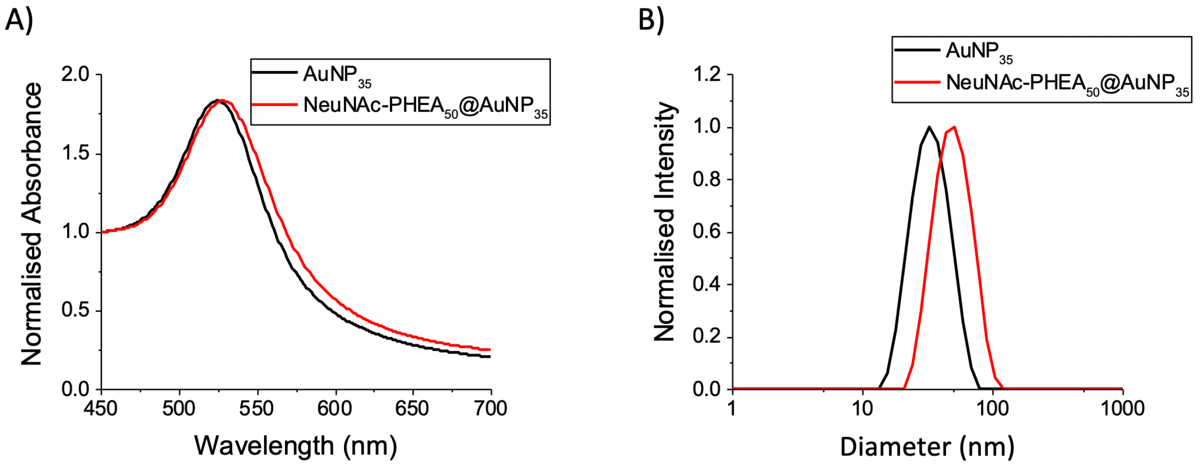

Figure S5. Characterisation of unfunctionalised and NeuNAc-functionalised 35 nm AuNPs by A) UV/Vis and B) dynamic light scattering.

Code	UVmax[a] (nm)	A_{SPR}/A_{450}[b]	D_h[c] (nm)	$D_{h\ (DLS)}$[d] (nm)	$D_{h\ (TEM)}$[e] (nm)
Citrate $AuNP_{35}$	526	1.91	35	34.5±1	35±3
NeuNAc-$PHEA_{50}AuNP_{35}$	531	1.99	45	55.3±1	38.9±3.1

Table S1. Characterization of unfunctionalised and functionalised AuNPs used in this study. (a) SPR absorption maximum; (b) Absorbance ratio of SPR to 450 nm; (c) Estimated from UV-Vis[4]; (d) From dynamic light scattering; (e) From TEM, from average of >100 particles, showing ±S.D.

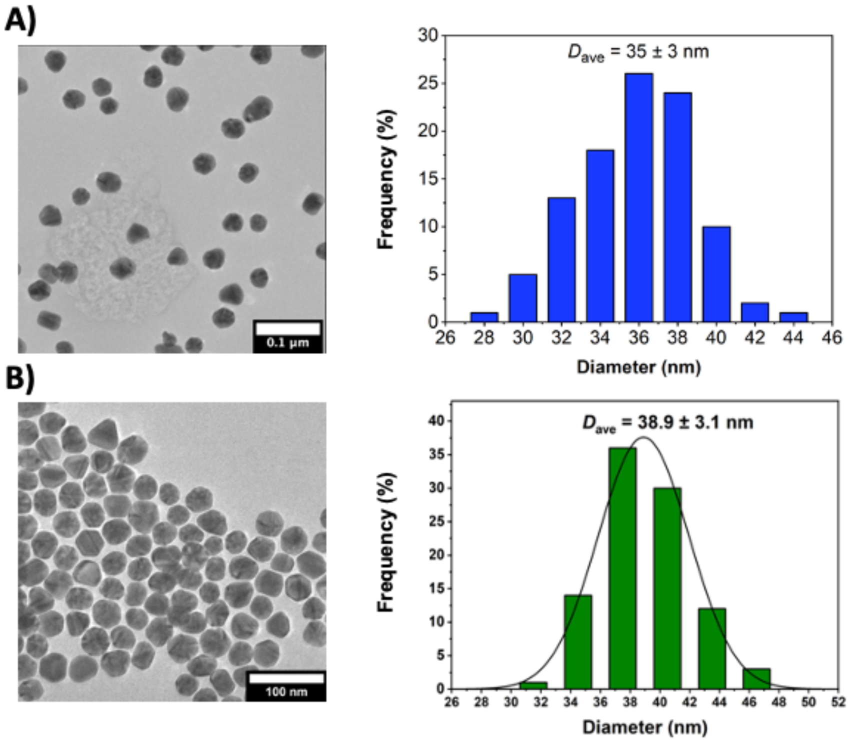

Figure S6. TEM images (left) and histograms (right) of citrate stabilized AuNPs. A) 35 nm unfunctionalised AuNP and B) NeuNAcPHEA$_{50}$@AuNP$_{35}$. Histograms from analysis of >100 particles

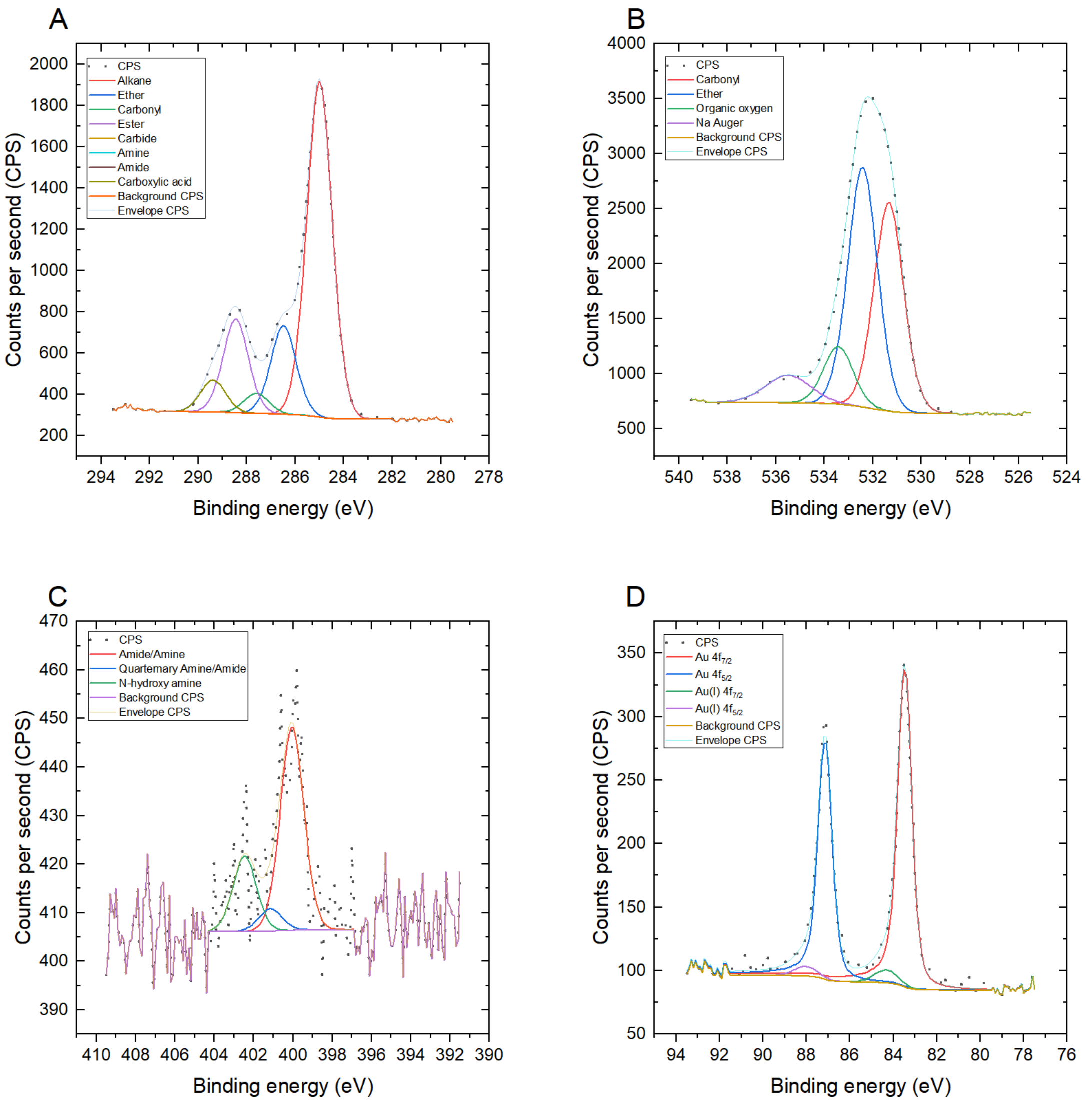

Figure S7. X-ray photo-electron spectroscopy (XPS) of citrate stabilized 35nm AuNP A) C 1*s* B) O 1*s* C) N 1*s* and D) Au 4*f*

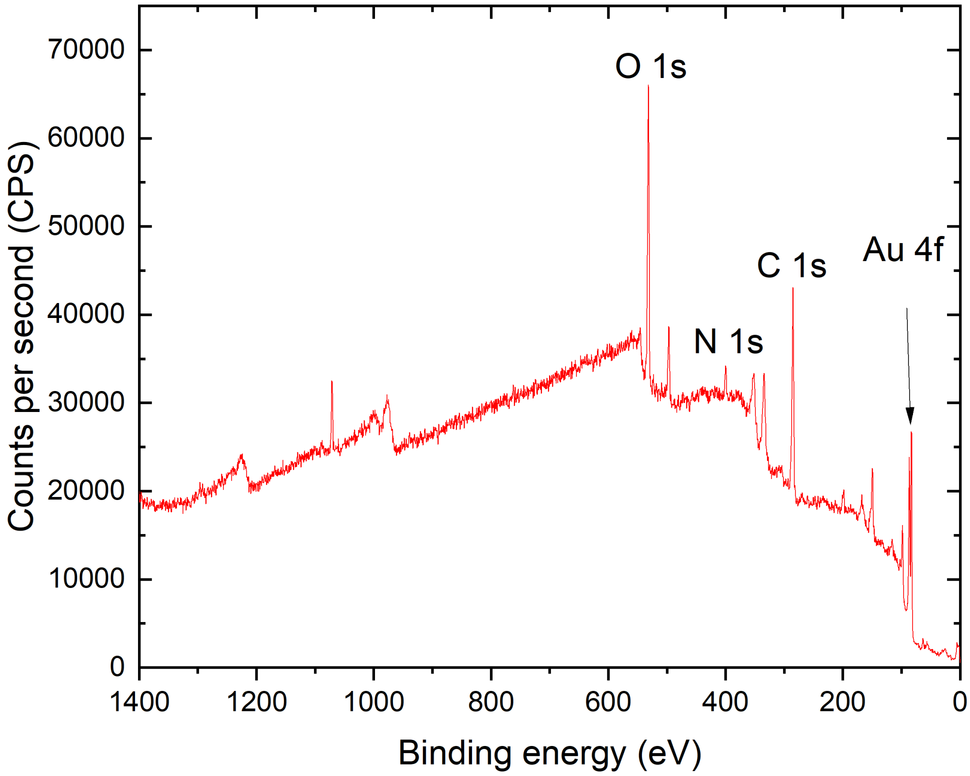

Figure S8. XPS survey scan of neuraminic acid $PHEA_{50}@AuNP_{35}$

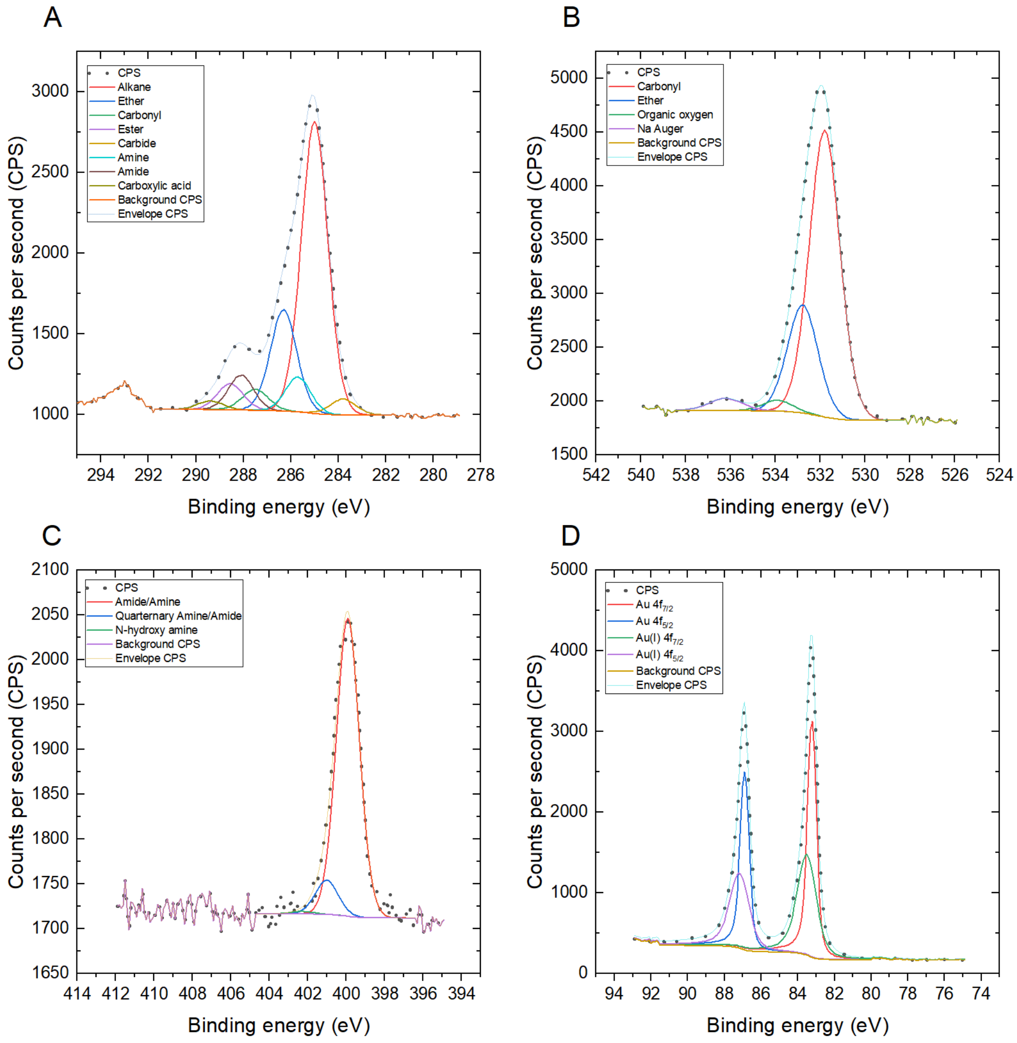

Figure S9. XPS of neuraminic acid $PHEA_{50}$@$AuNP_{35}$ A) C 1*s* B) O 1*s* C) N 1*s* and D) Au 4*f*

Characterization of 2-Azido-2-deoxy-N-acetyl-D-neuraminic acid

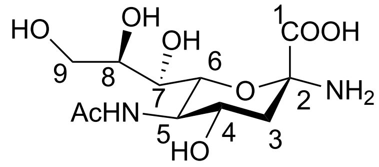

δ_H (400 MHz, D_2O) 4.05 - 3.89 (3H, m, H^4, H^5 and H^6), 3.83 (1H, dd, *J* 11.5, 1.5, H^{9a}), 3.78 - 3.71 (1H, m, H^8), 3.62 (1H, dd, *J* 11.5, 6.5, H^{9b}), 3.48 - 3.43 (1H, m, H^7), 2.13 - 2.00 (~5H, m, H^{3a}, H^{3b} and CH_3). δ_C NMR (400 MHz, D_2O) 174.6 (<u>C</u>OCH_3), 174.4 (C^1), 96.4 (C^2),70.7, 69.4 (C^6 and C^8), 67.8 (C^7) 66.0 (C^4), 63.2 (C^9), 53.3 (C^5), 38.6 (C^3), 22.0 (CH_3).

NB: The peaks at ~4.3 ppm and ~1.25 ppm in the ^{1}H NMR are TEA impurities

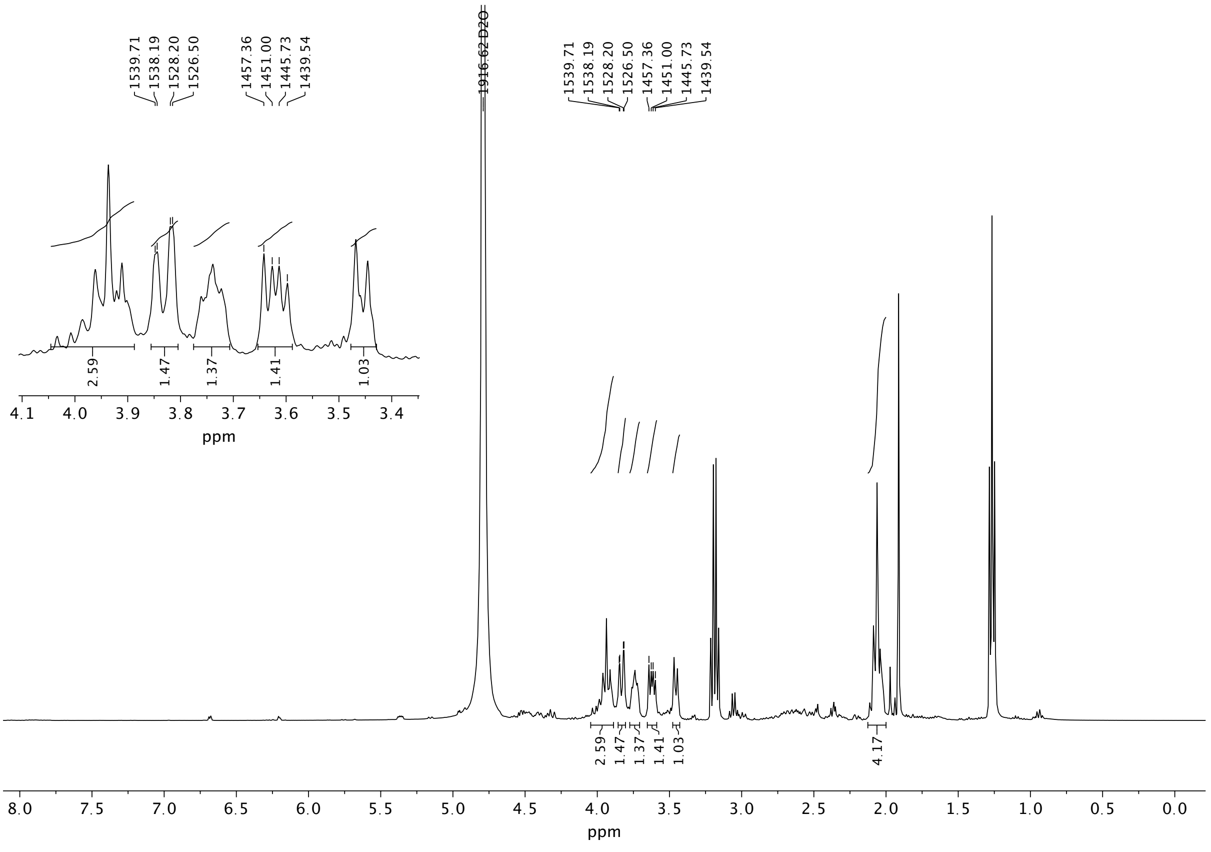

Figure S10. ^{1}H NMR of 2-amino-2-deoxy-*N*-acetyl-D-neuraminic acid

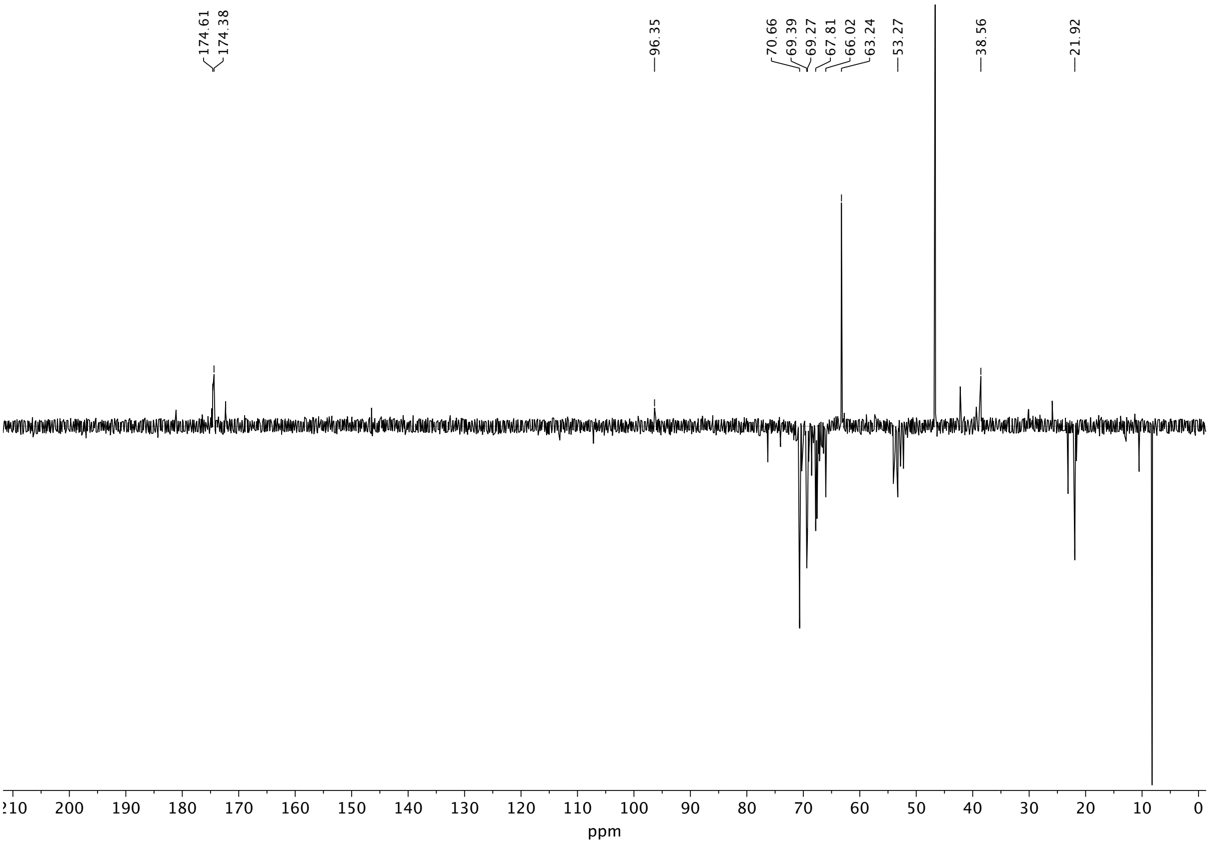

Figure S11. ^{13}C NMR of 2-amino-2-deoxy-*N*-acetyl-D-neuraminic acid

Recombinant Expression and Purification of truncated SARS-COV-2 Spike (S1) Protein (first 300 amino acids) in E.coli.

[All data reported in the manuscript/supporting information uses this protein, not that from HEK293 expression (detailed below), unless specified.]

A pET21a plasmid encoding for a hexahistidine-tag, SUMO-tag and the first 300 amino acids of SARS-COV-2 was purchased from Genscript Inc. The plasmid was transformed into competent *Escherichia coli* BL21(DE3) cells (New England Biolabs). A colony was selected to inoculate 100 mL of LB-medium containing 100 $\mu g.mL^{-1}$ kanamycin and was grown overnight at 37 °C under continuous shaking of 180 rpm. The following day, 10 mL of the preculture was added to 1 L of LB-medium (supplemented with 100 $\mu g.mL^{-1}$ kanamycin) in a 2.5 L Ultra Yield™ flask and grown at 37 °C with a shaking speed of 180 rpm till an OD_{600} of 0.6 was reached. The temperature was then reduced to 16 °C and the cells incubated for another hour before adding IPTG to a final concentration of 0.2 mM. The overexpression of the protein was allowed to take place overnight following which the cells were centrifuged at 5000 g for 10 minutes at 4 °C. Pelleted cells were resuspended in PBS supplemented with Pierce protease inhibitor mini-tablets. The suspension was passed through a STANSTED 'Pressure Cell' FPG12800 homogenizer in order to lyse the cells. The cell lysate was centrifuged at 48,000 g and the supernatant was passed through a 0.45 µm filter before being added to a 3 mL column of IMAC cOmplete His-Tag Purification Resin (Roche) pre-equilibrated with PBS. The column was washed with 20 column volumes of PBS. Bound protein was eluted using 6 mL of 300 mM Imidazole in PBS. Further purification of was achieved using a HiLoad 16/600 Superdex 200 pg gel-filtration column (GE Healthcare) with PBS as the running buffer. Purity was estimated using SDS-PAGE and protein concentration determined using Thermo Scientific Pierce BCA assay kit. Various volumes of the protein contained in PBS solution were aliquoted

into 1.5 mL microcentrifuge tubes and snap-frozen in liquid nitrogen to store at -80 °C until required.

Protein sequence expressed (N-terminal polyhistidine and SUMO tags with the first 300 amino acids of the spike protein);

MGSSHHHHHHGSGMSDSEVNQEAKPEVKPEVKPETHINLKVSDGSSEIFFKIKKTTPL
RRLMEAFAKRQGKEMDSLRFLYDGIRIQADQTPEDLDMEDNDIIEAHREQIGGGSEF
ELMFVFLVLLPLVSSQCVNLTTRTQLPPAYTNSFTRGVYYPDKVFRSSVLHSTQDLFL
PFFSNVTWFHAIHVSGTNGTKRFDNPVLPFNDGVYFASTEKSNIIRGWIFGTTLDSKT
QSLLIVNNATNVVIKVCEFQFCNDPFLGVYYHKNNKSWMESEFRVYSSANNCTFEY
VSQPFLMDLEGKQGNFKNLREFVFKNIDGYFKIYSKHTPINLVRDLPQGFSALEPLVD
LPIGINITRFQTLLALHRSYLTPGDSSSGWTAGAAAYYVGYLQPRTFLLKYNENGTIT
DAVDCALDPLSETK

NB: The 300 amino acids of the spike protein are underlined

SARS-COV-2 spike protein variants

In order to establish whether the glycan-flow through concept was capable of detecting new variants of SARS-COV-2, a number of truncated recombinant spike proteins containing mutations associated with SARS-COV-2 variants were expressed (in *E. coli*). The plasmids encoding the variants were purchased from Genscript Inc and expressed using the above protocol (entitled *Recombinant Expression and Purification of truncated SARS-COV-2 Spike (S1) Protein (first 300 amino acids) in E.coli).*

**First detection location**	**PANGO lineage**	**Relevant mutations**
Brazil	P.1	L18F, T20N, P26S, D138Y, R190S
United Kingdom	B.1.1.7	H69-V70 deletion, Y144 deletion
South Africa	B.1.351	L18F, D80A, D215G, R246I

Expression and purification of SARS-COV-2 Spike (S1) in HEK293 Cells

[reproduced from[1] where we previously expressed this protein]

Codon-optimised SARS-COV-2 Spike (S1) subunit (amino acids 1-685) with a C-terminal 10x polyhistidine tag was expressed under control of a CMV promoter (pCMV3-S1-10xHis, Sino Biological, #VG40591-CH). HEK293 cells were grown in suspension to a density of 1.0 x 10^6 cells/mL in FreeStyle 293 Expression Medium (Thermo Scientific, #12338018), then transfected with 0.5 µg of pCMV3-S1-10xHis, 1.5 µg of linear polyethyleneimine (Alfa Aesar, #43896.01) and 50 µL Opti-MEM-I per 1 mL of cells (Thermo Scientific, #31985-062). After transfection, cells were grown to a density of 2.0 x 10^6 cells/mL and supplemented with 4 mM valproic acid (Sigma Aldrich, #P4543). 96 hours post transfection, the media was cleared by centrifugation, 6,000 x g in a Fiberlite F10-4 x 1000 LEX rotor (Thermo Scientific, #096-041053) for 10 minutes.

To purify Spike S1, cleared media was adjusted to 20 mM HEPES pH 7.5 and 10 mM imidazole, and was loaded on to a 5 mL HisTrap HP column (cytiva, #17524801) at a flow rate of 20 mL/min for ~16 hours. A purification buffer comprising 20 mM HEPES, 300 mM NaCl and 1 mM DTT +/- 1 M imidazole was used (for buffer lines A and B respectively), and the column was washed with 30 CVs of 2% buffer B (20 mM imidazole) before eluting the protein over a 2-50% gradient over 30 CVs. Fractions containing Spike (S1) were pooled and concentrated using a 10 KDa molecular-weight cut-off spin concentrator (Sigma Aldrich, #UFC910008), before being buffer exchanged into 20 mM HEPES 7.5, 300 mM NaCl, 10% glycerol using a 5 mL HiTrap desalting column (cytiva, #29048684). Peak fractions were pooled, and the final concentration was measured by absorbance at 280 nm, yielding a concentration of 1.25 mg/mL. Aliquots of protein were snap-frozen in liquid nitrogen and stored at -80°C.

The protein produced by this method (*Expression and purification of SARS-COV-2 Spike (S1) in HEK293 Cells*) was used to check new gold nanoparticle batches and cassettes produced during the initial development of this work and for the Tergitol experiments (Figure 2C, main paper). *E.coli* expressed spike protein was used for all other analysis, as this enabled variant sequences to be expressed, ensuring consistency.

Flow-Through Cassette Production, Running and Analysis Protocols

Flow-Through Cassette Production, Running and Analysis Protocols

The following Flow-through Cassette buffers have been previously reported,[1] but have been provided for the reader. Minor optimisations have been made to the other protocols, so the full procedure has been provided for clarity.

Protocol for Manufacturing Flow-Through Cassettes

Nitrocellulose was added to the backing card by attaching the plastic backing of the nitrocellulose to the self-adhesive on the card. The wick material was then added to the backing card so it overlaps with the nitrocellulose by ~5 mm. The strips were then cut to size of width ~3 mm so they sit in the cassettes without the need for excess force to fit. The conjugate pad was added to the backing card, so it overlaps with the nitrocellulose by ~3.5 mm. The sample pad, was cut to size of 20 mm by 6 mm and was added to the backing card, overlapping with the conjugate pad by ~6.5 mm and straddling the backing card evenly. The completed strip was then added to the cassettes and sealed.

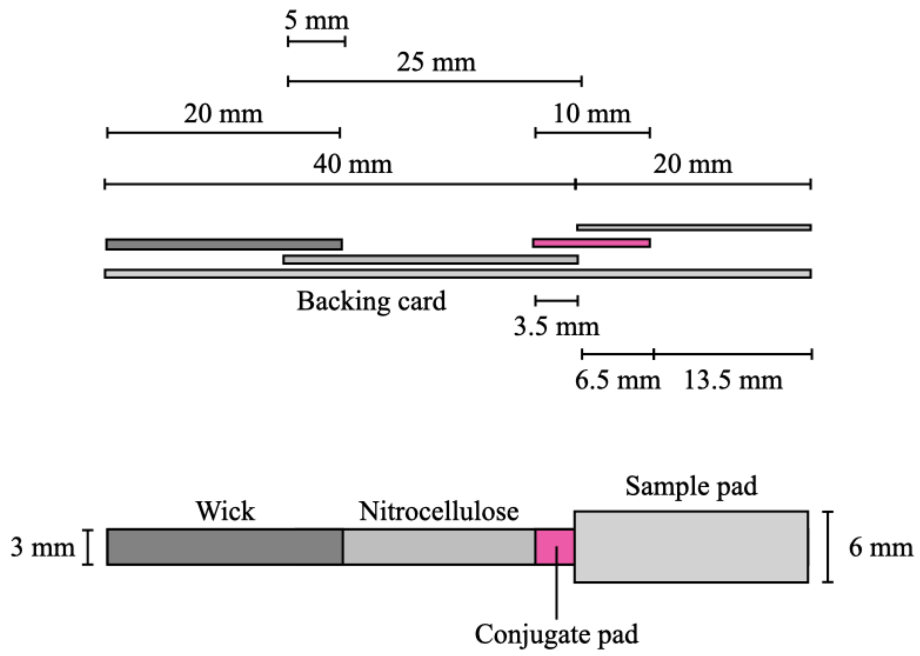

Figure S12. Flow-through complete strip dimensions

Protocol for Conjugate Pad Production

Strips of the conjugate pad material were agitated for 30 minutes in a solution of 0.1% Tween-20 (blocking solution). The strips were then patted dry and baked overnight at 37 °C in an oven. The conjugate pads were cut to size (3 mm width) and placed individually into the wells of a 384-well microplate. 20 µL 1× conjugate pad buffer solution containing OD3 (unless otherwise specified) AuNPs was added to the top of each conjugate pad in the wells. The pads were dried overnight at 37 °C in an oven. The completed pads were stored in an airtight box containing desiccant until addition to the strips.

10× Conjugate Pad Buffer

10% w/v. of poly(vinyl pyrrolidone)$_{400}$ (Average Mw ~40,000 g.mol^{-1}), 50% w/v. trehalose, 10% w/v. sucrose and 0.1% w/v. Tween-20 were added to distilled water and allowed to dissolve.

Control Line Addition

Control lines of 1 μL of RCA_{120} were added to the nitrocellulose strips using a micropipette fitted with a 10 μL tip. A control line was added ~1.5 cm from the non-wick end of the nitrocellulose surface. The strips were dried at 37 °C in an oven for 30 minutes.

Sample Line Addition

Sample lines of 1 μL were added to the nitrocellulose strip using a micropipette fitted with a 10 μL tip, the sample was spotted ~1 cm from the non-wick end of the nitrocellulose surface. The strips were dried at 37 °C in an oven as described in the figures.

Protocol for running flow-through tests

10 μL 10× HEPES buffer (20% PVP_{400}) was added to 90 μL distilled water. 100 μL was added to the cassette well and allowed to absorb. The test was run for X minutes, as described in the figures, before photos were taken.

Silver Staining Procedure

Staining solution was prepared following the kit guidelines. 50 μL of solution A and 50 μL of solution B were mixed and added to the cassette well. The test was run for X minutes, as described in the figures, before photos were taken.

Flow-through assay buffer - 10× HEPES buffer (20% PVP_{400}) in 100 mL H_2O

2.38 g (100 $mmoldm^{-3}$) of HEPES, 8.77 g (1.50 $moldm^{-3}$) of NaCl, 0.011 g (1.0 $mmoldm^{-3}$) of $CaCl_2$, 0.8 g (0.8% w/v., 123 $mmoldm^{-3}$) of NaN_3, 0.5 g (0.5% w/v., 4.07 $mmoldm^{-3}$) of Tween-20 and 20 g (20% w/v.) of poly(vinyl pyrrolidone)$_{400}$ (PVP_{400}, Average Mw ~40,000) was dissolved in 100 mL of water. The buffer is not pH adjusted.

Protocol for analysing flow-through tests to determine signal intensity and intensity change

Images collected were analysed in Image J 1.51 using the plot profile function to create a data set exported to Microsoft Excel for Mac. The data was exported to Origin 2019 64Bit and trimmed to remove pixel data not from the strip surface. The data was then reduced by number of groups to 100 data points (just the nitrocellulose surface) and plotted as Grey value (scale) vs Relative distance along the 100 data points.

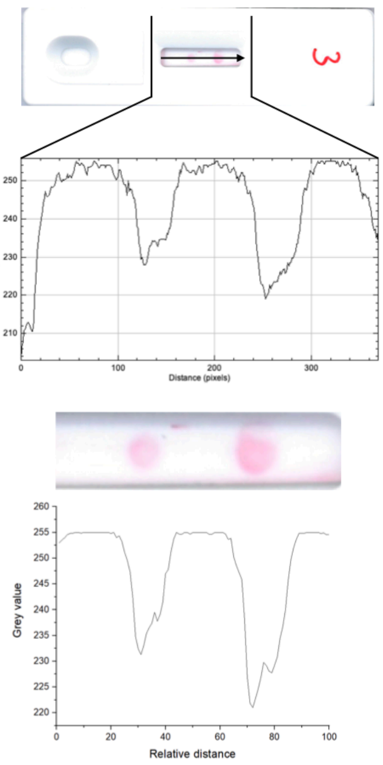

Figure S13. Representative cassette (Top), raw grey value plot (Middle) and processed grey value plot (Bottom)

Relative distance pixel 1 to 50 and 51 to 100 (area around the test line and control line, respectively), excluding pixels that contributed to the signal peaks were averaged (mean). This average was subtracted from the lowest grey value between 1 to 50 (test spot) and 51 to 100 (control spot) respectively. To determine the change after silver staining, the signal intensity before and after silver staining was calculated and subtracted from one another to give Intensity Change (Figure 4D).

Additional Data and Figures

Flow-Through Strips and Plotted Data

None of the images in this supplementary information have been imaged enhanced. The flow charts below the tables describe the testing process for the cassette tables above (this does not include control line addition). It should be noted that the negative swab samples used were arbitrarily numbered one to x for each batch of swab samples used. Therefore, negative samples in different tables are from different swab sample batches, so are independent samples from different patients to those in other tables.

Cassettes from Pseudotyped Lentivirus Experiments

Titre, TU/ml	Particles	Cassettes after 20 minute run
1.5 × 10⁵	NeuNAc-PHEA50AuNP35	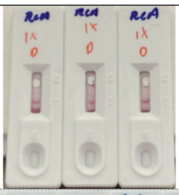
1.5 × 10⁴	NeuNAc-PHEA50AuNP35	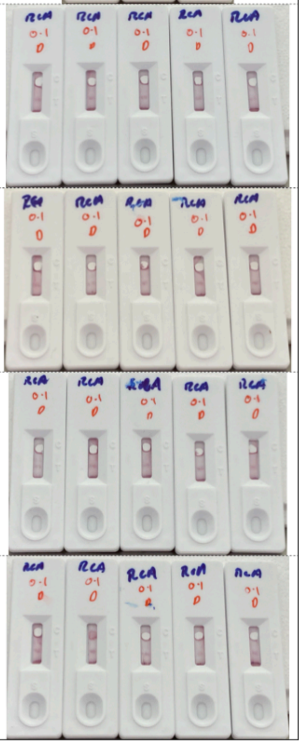

Table S2. Photos of flow-through cassettes versus a sample line of heat deactivated Spike (SARS-COV2) pseudotyped lentivirus at varying concentrations. Titre concentration was measured in transduction units per millilitre (TU/mL). A control line of 5 mg/mL RCA_{120} was used, which gave rise to the 'white' control line, later optimized.

1 µL sample added → Dried for 30 minutes at 37 °C → 100 µL buffer added → Picture taken after 20 minutes

Titre, TU/ml	Particles	Cassettes after 20 minute run
1.5×10^4	NeuNAc-PHEA50AuNP35	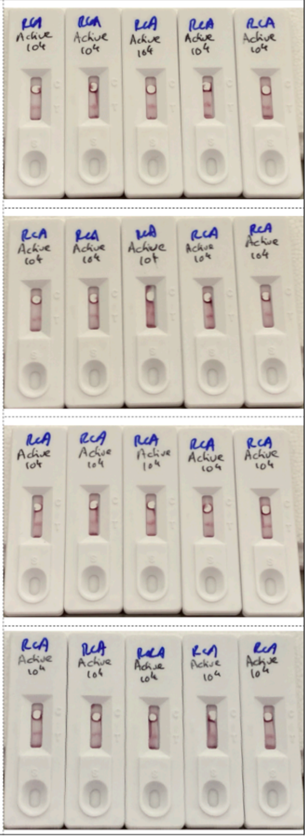

Table S3. Photos of repeat flow-through cassettes versus a sample line of Spike (SARS-COV2) pseudotyped lentivirus at 1.5×10^4 TU/mL. Titre concentration was measured in transduction units per millilitre (TU/mL). A control line of 5 mg/mL RCA_{120} was used, which gave rise to the 'white' control line, later optimized.

1 µL sample added → Dried for 30 minutes at 37 °C → 100 µL buffer added → Picture taken after 20 minutes

Titre, LP/mL	Particles	Cassettes after 20 minute run
~1 × 10^9	NeuNAc-PHEA50AuNP35	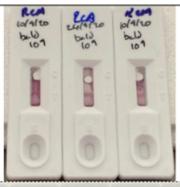
~1 × 10^4	NeuNAc-PHEA50AuNP35	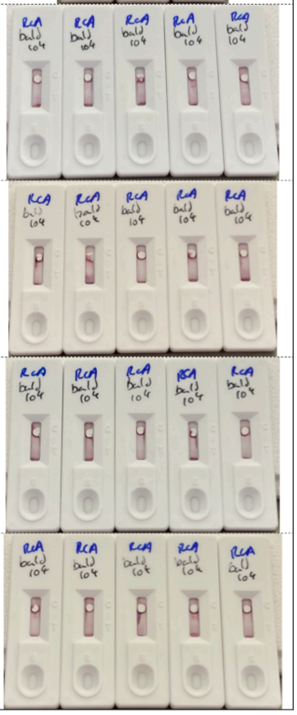

Table S4. Photos of flow-through cassettes versus a sample line of bald pseudotyped lentivirus at varying concentrations. Titre concentration was measured in lentiviral particles per millilitre (LP/mL). A control line of 5 mg/mL RCA_{120} was used, which gave rise to the 'white' control line, later optimized.

1 µL sample added → Dried for 30 minutes at 37 °C → 100 µL buffer added → Picture taken after 20 minutes

Titre	Particles	Time since cassette manufacture, days	Cassettes after 20 minute run
Active, 1.5×10^4	NeuNAc-PHEA50AuNP35	21	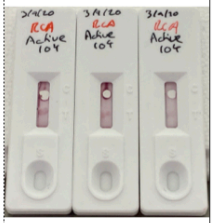
Bald, $\sim 1 \times 10^4$	NeuNAc-PHEA50AuNP35	21	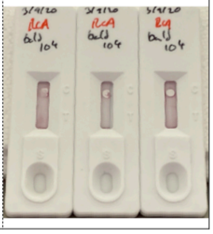

Table S5. Photos of old flow-through cassettes, stored at RTP and open to the atmosphere for 21 days post-production before use, versus a sample line of differing lentiviruses. Titre concentration was measured in transduction units per millilitre (TU/mL) for the Spike (SARS-COV2) pseudotyped lentivirus ("Active") and in lentiviral particles per millilitre (LP/mL) for the bald pseudotyped lentivirus ("Bald"). A control line of 5 mg/mL RCA_{120} was used giving the 'white' background. Control line optimization is discussed in the main text.

1 µL sample added → Dried for 30 minutes at 37 °C → 100 µL buffer added → Picture taken after 20 minutes

Cassettes from Swab Sample Experiments <u>Not Used</u> to Determine Specificity and Selectivity

Tables S6/7 shows samples with swabs used to evaluate the impact of volume of sample spotted to the test line and not used in the sensitivity and specificity testing.

Ct value	Number of times sample spotted as test line	After 5 minutes	After 20 minutes
8.3	1	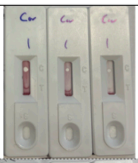	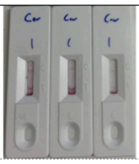
8.3	2	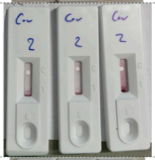	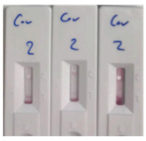
8.3	3	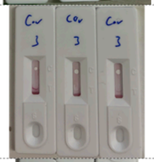	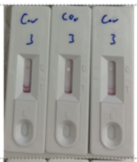
8.3	4	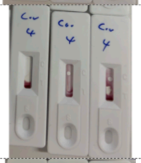	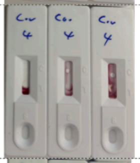
8.3	5	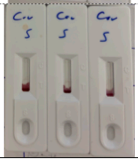	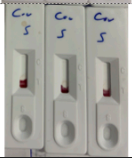

Table S6. Photos of flow-through cassettes versus sample lines of a swab sample of known Ct value as a function of total volume of sample applied. A 1 µL sample volume was added ("spotted") once or multiple times. A control line of 5 mg/mL RCA_{120} was used.

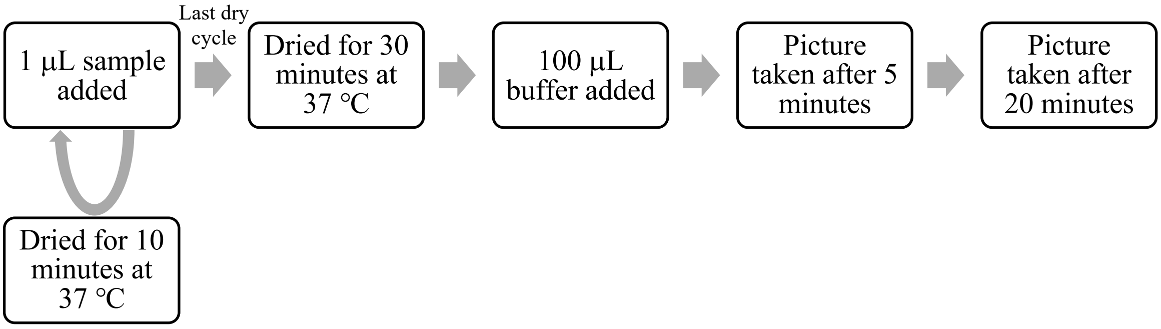

Ct value	Number of times sample spotted as test line	After 5 minutes	After 20 minutes
Negative	1	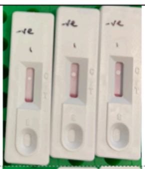	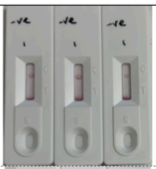
Negative	2	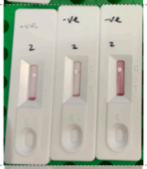	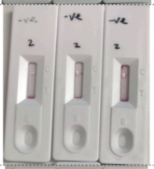
Negative	3	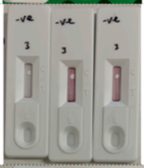	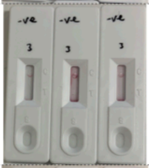
Negative	4	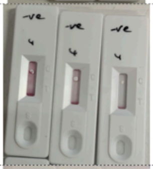	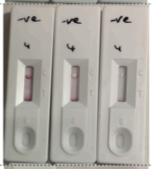
Negative	5	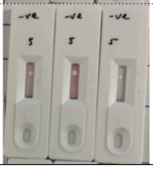	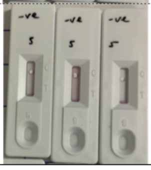

Table S7. Photos of flow-through cassettes versus sample lines of a negative swab sample. A 1 µL sample volume was added ("spotted") once or multiple times. A control line of 5 mg/mL RCA_{120} was used, which was not optimized and is discussed in the main text.

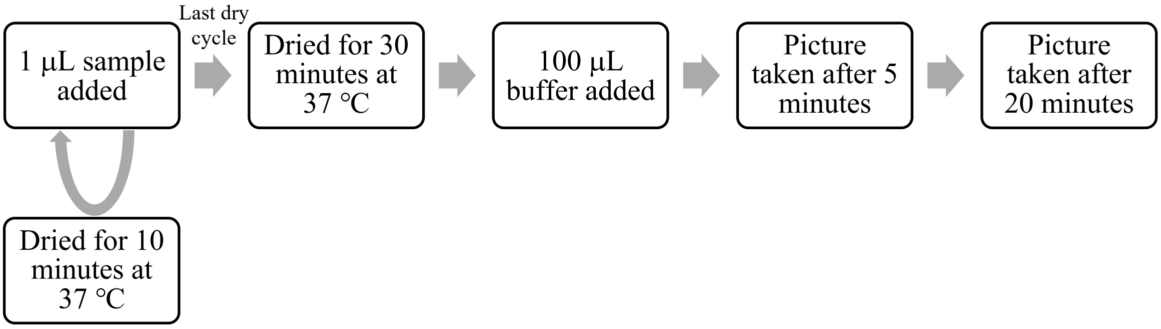

Ct value	Cassette 50 mins after 2nd buffer	Cassette after 20 min silver stain	Ct value	Cassette 50 mins after 2nd buffer	Cassette after 20 min silver stain	Ct value	Cassette 50 mins after 2nd buffer	Cassette after 20 min silver stain
16.76	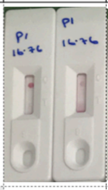	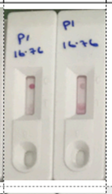	22.59	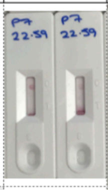	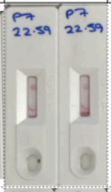	11.03	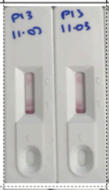	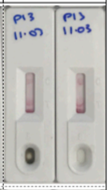
10.23	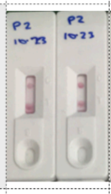	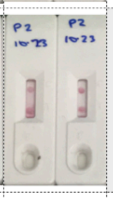	9.42	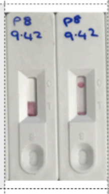	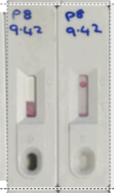	11.38	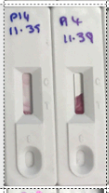	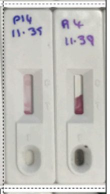
15.25	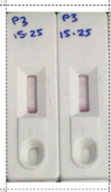	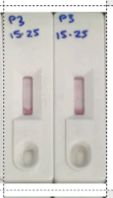	9.38	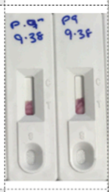	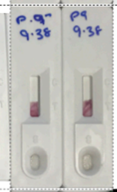	10.06	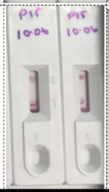	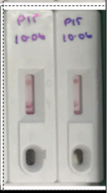
9.77	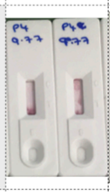	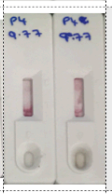	6.29	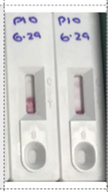	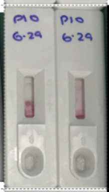			
9.93	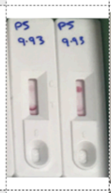	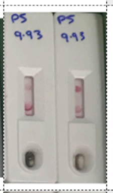	5.99	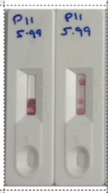	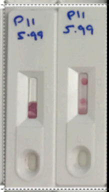			
21.70	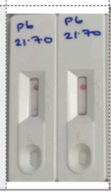	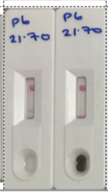	9.16	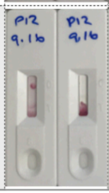	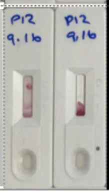			

Table S8. Photos of flow-through cassettes versus sample lines of swab samples of known Ct values showing impact of over-loading sample. A 1 μL sample volume was added ("spotted") three times, with sample over-loading clear. A control line of 1 mg/mL RCA_{120} was used.

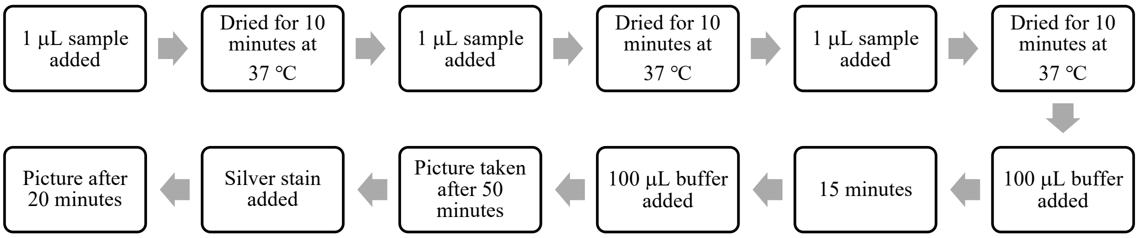

Ct value	Heat stressed	Cassette 15 mins after 1st buffer	Cassette 15 mins after 2nd buffer
6.29	n/a	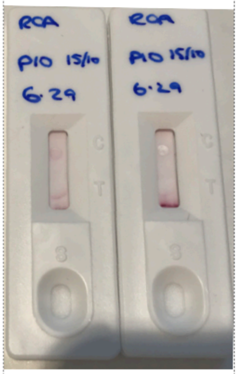	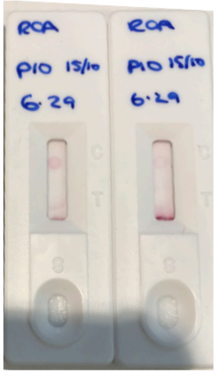
6.29	70°C for 12 hours	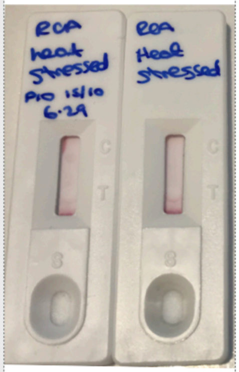	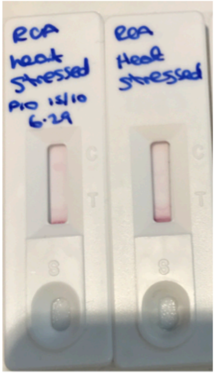

Table S9. Photos of flow-through cassettes put through different conditions versus sample lines of a positive swab sample. A 1 µL sample volume was added ("spotted") twice. A control line of 1 mg/mL RCA_{120} was used. Test line was visible in all (as crescent), but photograph is quite exposed [but no image enhancements are used here].

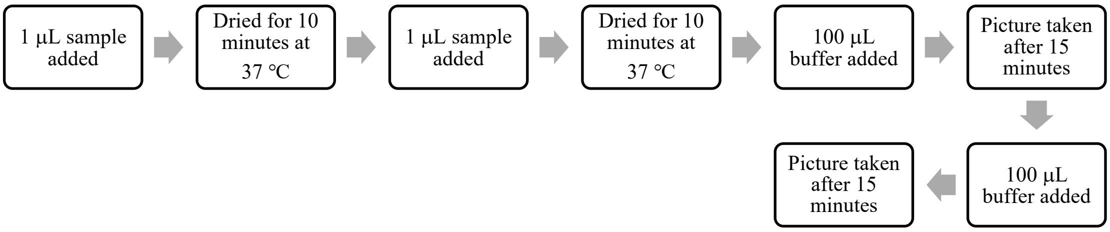

NB: Cassettes that were heat stressed had the control line added prior to heating at 70 °C for 12 hours.

Cassettes from Swab Sample Experiments Used to Determine Specificity and Selectivity

Cassettes used to produce the confusion tables to assess the prototype diagnostic's performance have been labeled with; "+" for a test judged to give a positive result, "–" for a test judged to give a negative result and "F" for a test that failed to run. Failed tests are excluded from calculations but are reported. Unlike in previous tests, the control line was always 1 mg/mL RCA_{120}. In silver stained cassettes control lines were more robust (as this was a non-optimized component) and hence in main paper only silver-stained performance measurement is shown. However, guided by controls in silver, the performance of non-stained was also predicted, to allow the impact of staining on overall performance to be discussed, and is plotted below.

Ct value	Cassette 15 mins after 2nd buffer	Cassette after 20 min silver stain	Ct value	Cassette 15 mins after 2nd buffer	Cassette after 20 min silver stain	Ct value	Cassette 15 mins after 2nd buffer	Cassette after 20 min silver stain
16.76	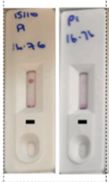	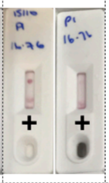	22.59	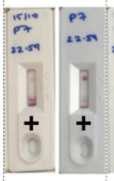	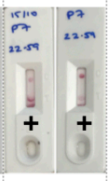	11.03	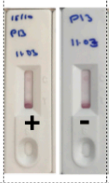	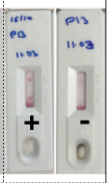
10.23	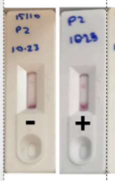	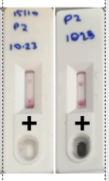	9.42	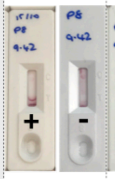	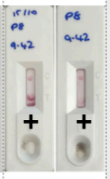	11.38	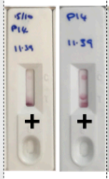	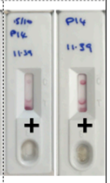
15.25	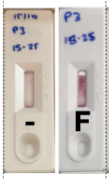	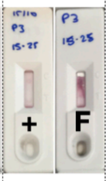	9.38	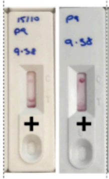	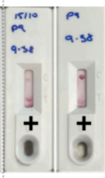	10.06	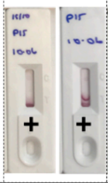	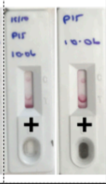
9.77	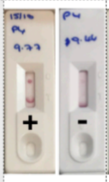	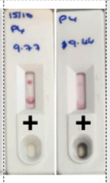	6.29	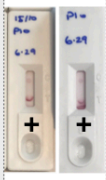	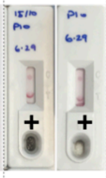			
9.93	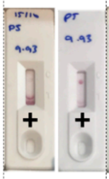	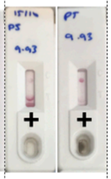	5.99	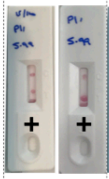	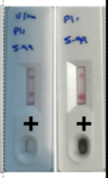			
21.70	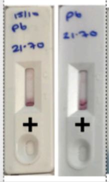	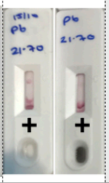	9.16	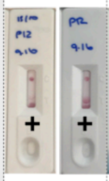	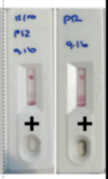			

Table S10. Photos of flow-through cassettes versus sample lines of swab samples of known Ct values. A 1 μL sample volume was added ("spotted") twice. A control line of 1 mg/mL RCA_{120} was used.

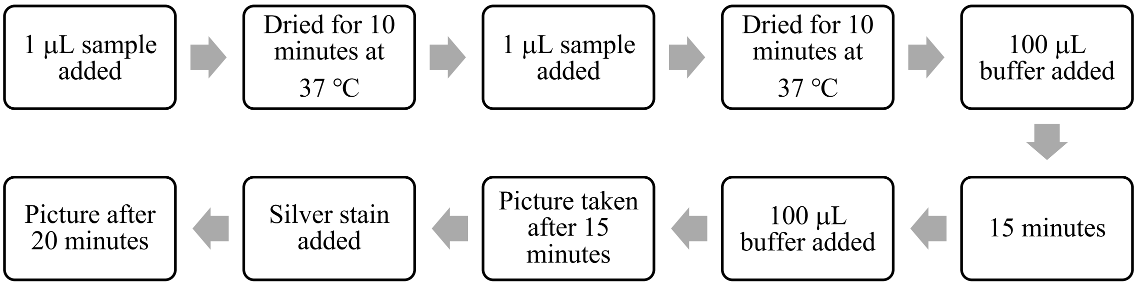

Ct value	Cassette 15 mins after 2nd buffer	Cassette after 20 min silver stain	Ct value	Cassette 15 mins after 2nd buffer	Cassette after 20 min silver stain	Ct value	Cassette 15 mins after 2nd buffer	Cassette after 20 min silver stain
11.03	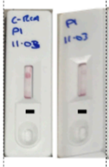	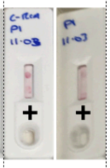	22.23	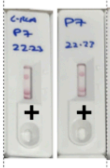	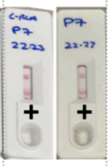	11.29	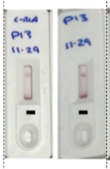	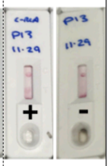
11.39	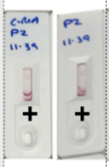	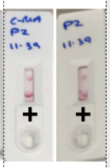	14.99	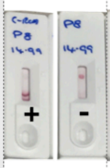	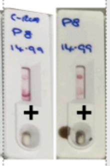	25.25	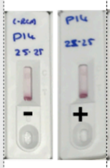	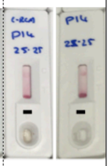
10.06	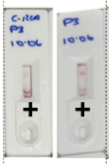	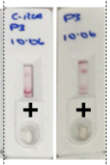	7.94	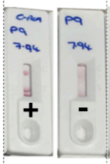	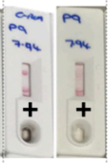	15.31	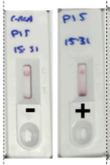	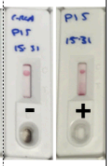
9.87	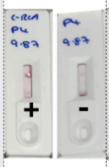	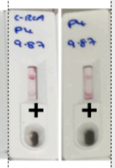	12.17	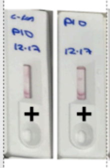	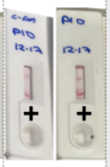	15.95	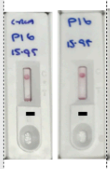	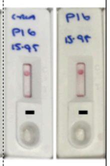
12.82	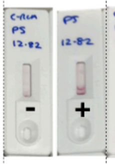	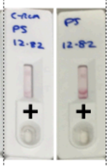	25.27	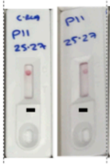	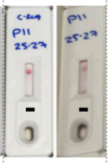	19.07	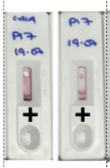	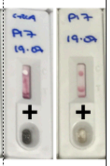
22.25	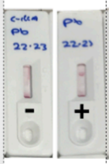	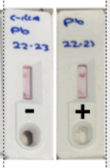	20.82	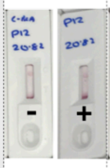	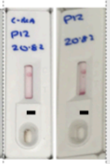	24.55	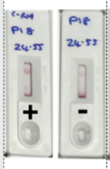	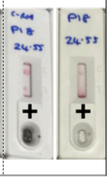

Table S11. Photos of flow-through cassettes versus sample lines of swab samples of known Ct values. A 1 μL sample volume was added ("spotted") twice. A control line of 1 mg/mL RCA_{120} was used.

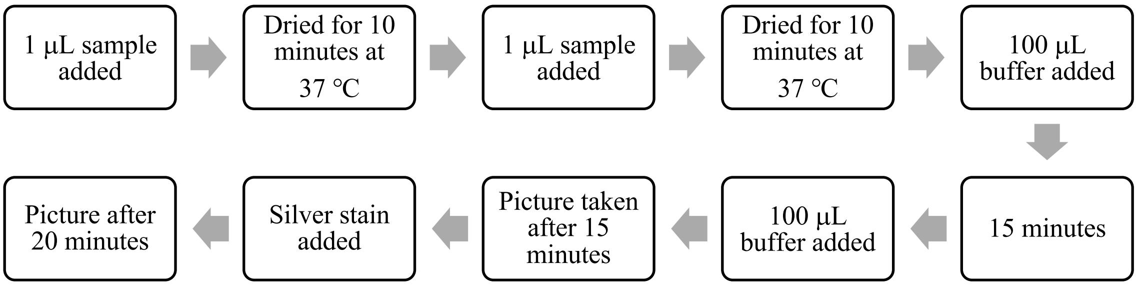

Ct value	Cassette 15 mins after 2nd buffer	Cassette after 20 min silver stain	Ct value	Cassette 15 mins after 2nd buffer	Cassette after 20 min silver stain	Ct value	Cassette 15 mins after 2nd buffer	Cassette after 20 min silver stain
17.63	- -	- -	7.03	+ +	+ +	7.38	+ -	+ +
7.74	+ +	+ +	7.31	+ +	+ +	9.46	+ +	+ +
16.80	- +	- +	6.92	+ +	+ +	7.62	+ +	+ +
9.64	+ +	+ +	7.36	+ -	+ +			
6.33	+ +	+ +	11.23	+ -	+ -			
13.02	- +	+ +	8.21	+ +	+ +			
14.34	+ +	+ +	23.42	+ +	+ +			

Table S12. Photos of flow-through cassettes versus sample lines of swab samples of known Ct values. A 1 µL sample volume was added ("spotted") twice. A control line of 1 mg/mL RCA_{120} was used.

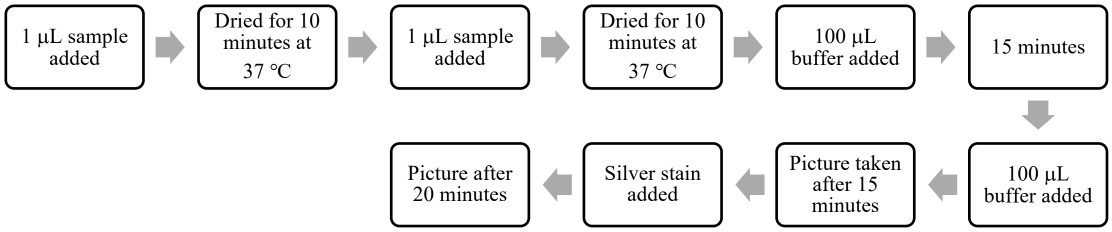

Ct value	Cassette 15 mins after 2nd buffer	Cassette after 20 min silver stain	Ct value	Cassette 15 mins after 2nd buffer	Cassette after 20 min silver stain	Ct value	Cassette 15 mins after 2nd buffer	Cassette after 20 min silver stain
Negative 1	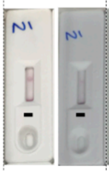	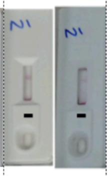	Negative 7	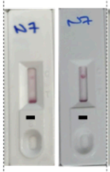	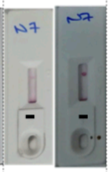	Negative 13	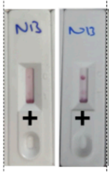	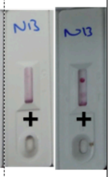
Negative 2	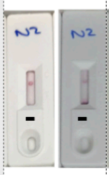	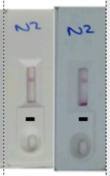	Negative 8	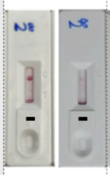	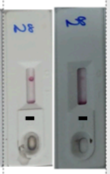	Negative 14	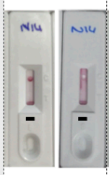	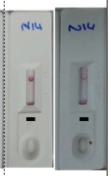
Negative 3	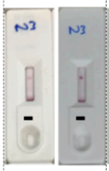	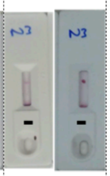	Negative 9	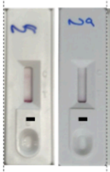	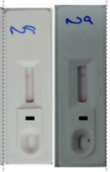	Negative 15	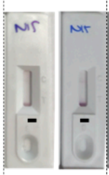	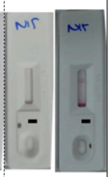
Negative 4	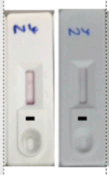	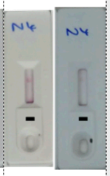	Negative 10	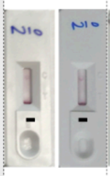	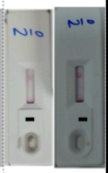			
Negative 5	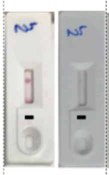	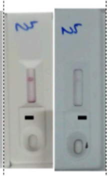	Negative 11	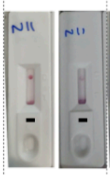	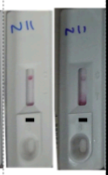			
Negative 6	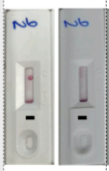	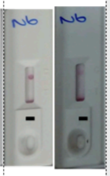	Negative 12	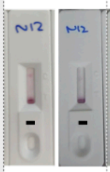	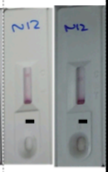			

Table S13. Photos of flow-through cassettes versus sample lines of negative swab samples. A 1 µL sample volume was added ("spotted") twice. A control line of 1 mg/mL RCA_{120} was used.

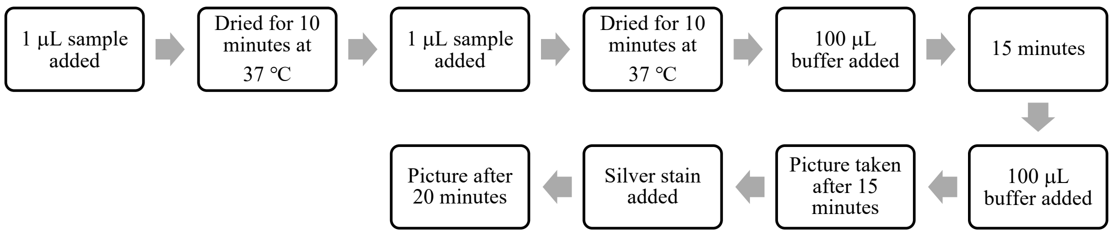

Ct value	Cassette 15 mins after 2nd buffer	Cassette after 20 min silver stain	Ct value	Cassette 15 mins after 2nd buffer	Cassette after 20 min silver stain	Ct value	Cassette 15 mins after 2nd buffer	Cassette after 20 min silver stain
Negative 1	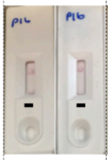	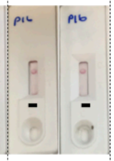	Negative 7	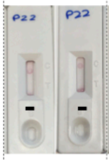	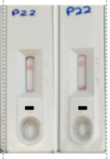	Negative 13	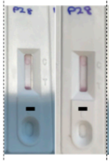	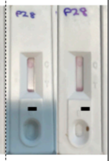
Negative 2	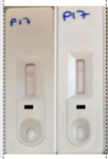	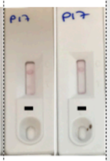	Negative 8	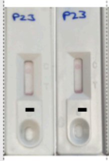	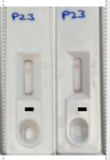	Negative 14	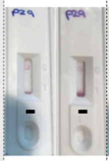	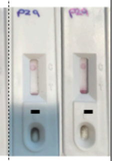
Negative 3	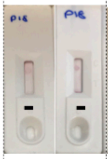	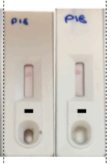	Negative 9	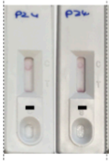	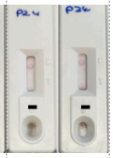	Negative 15	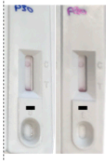	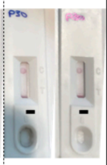
Negative 4	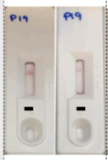	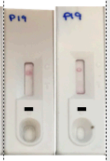	Negative 10	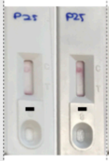	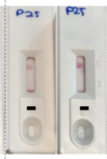			
Negative 5	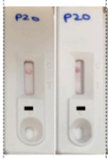	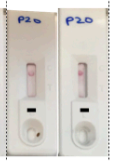	Negative 11	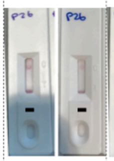	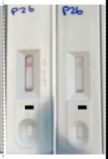			
Negative 6	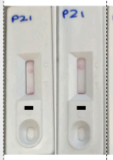	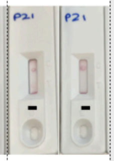	Negative 12	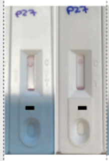	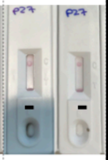			

Table S14. Photos of flow-through cassettes versus sample lines of negative swab samples. A 1 µL sample volume was added ("spotted") twice. A control line of 1 mg/mL RCA_{120} was used.

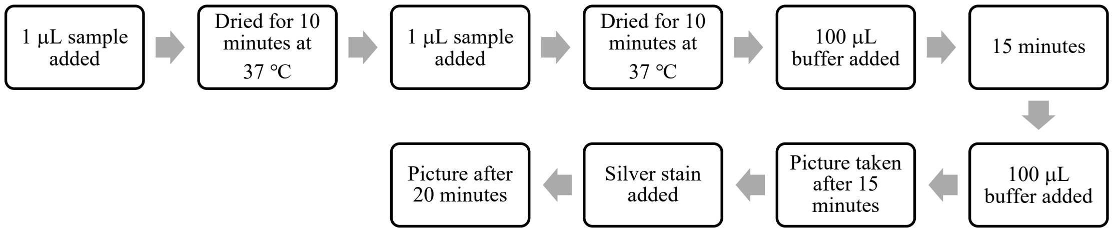

Ct value	Cassette 15 mins after 2nd buffer	Cassette after 20 min silver stain	Ct value	Cassette 15 mins after 2nd buffer	Cassette after 20 min silver stain	Ct value	Cassette 15 mins after 2nd buffer	Cassette after 20 min silver stain
Negative 1	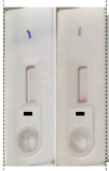	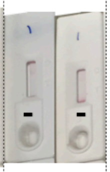	Negative 7	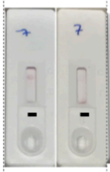	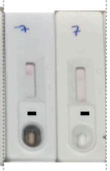	Negative 13	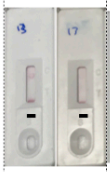	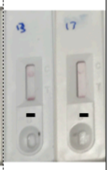
Negative 2	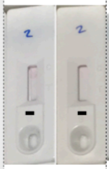	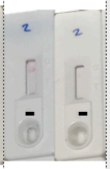	Negative 8	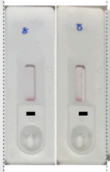	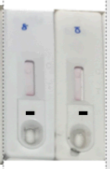	Negative 14	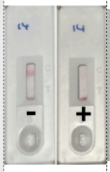	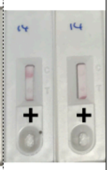
Negative 3	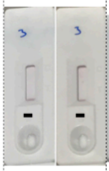	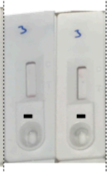	Negative 9	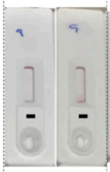	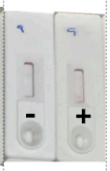	Negative 15	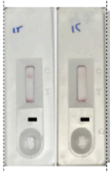	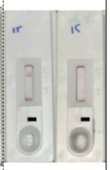
Negative 4	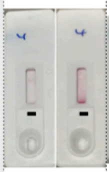	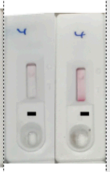	Negative 10	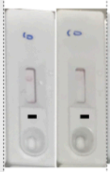	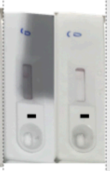	Negative 16	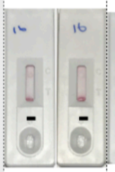	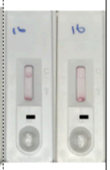
Negative 5	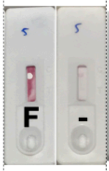	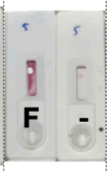	Negative 11	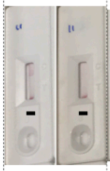	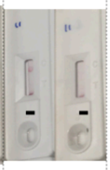	Negative 17	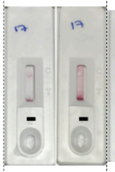	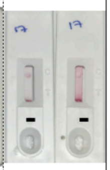
Negative 6	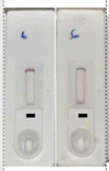	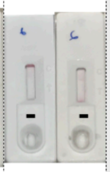	Negative 12	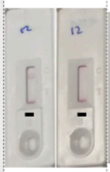	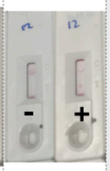	Negative 18	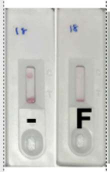	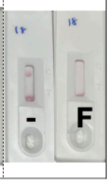

Table S15a. Photos of flow-through cassettes versus sample lines of negative swab samples. A 1 µL sample volume was added ("spotted") twice. A control line of 1 mg/mL RCA_{120} was used.

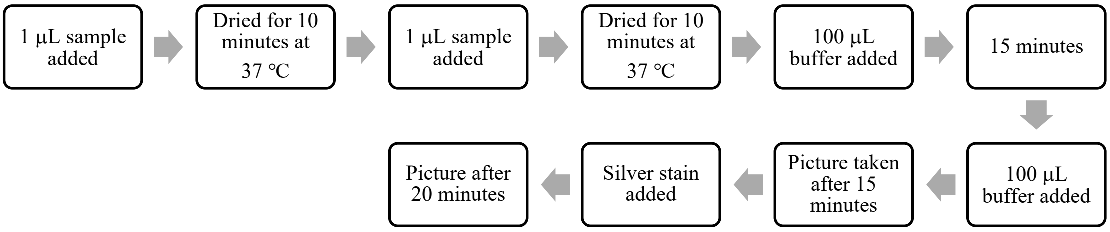

Ct value	Cassette 15 mins after 2nd buffer	Cassette after 20 min silver stain	Ct value	Cassette 15 mins after 2nd buffer	Cassette after 20 min silver stain	Ct value	Cassette 15 mins after 2nd buffer	Cassette after 20 min silver stain
Negative 19	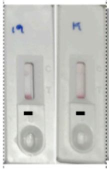	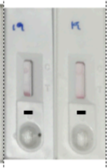	Negative 25	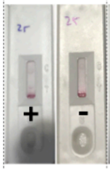	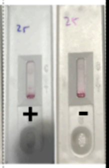			
Negative 20	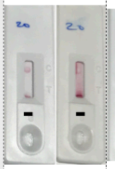	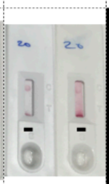						
Negative 21	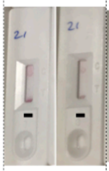	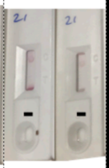						
Negative 22	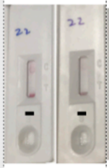	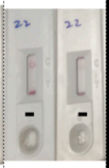						
Negative 23	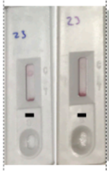	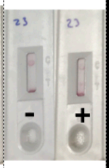						
Negative 24	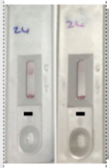	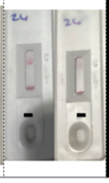						

Table S15b. Photos of flow-through cassettes versus sample lines of negative swab samples. A 1 µL sample volume was added ("spotted") twice. A control line of 1 mg/mL RCA_{120} was used.

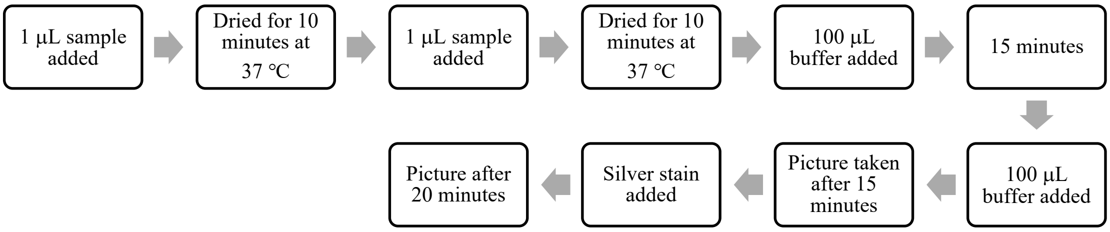

Analysis of Patient Samples Before Silver Staining

A)

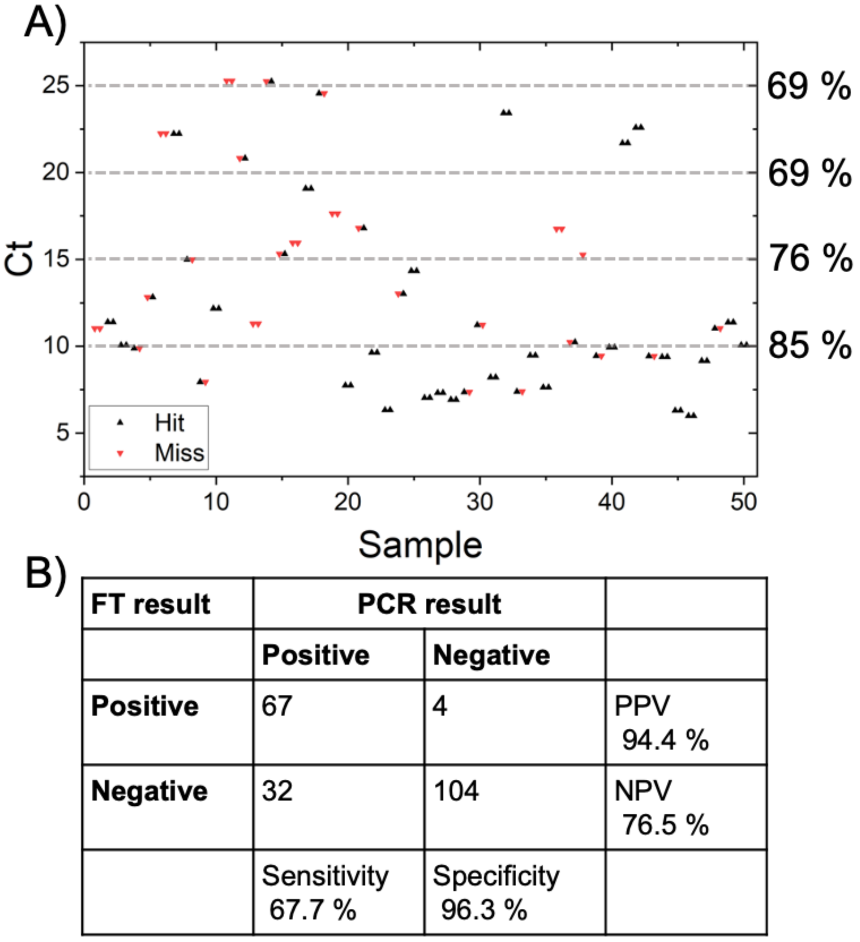

B)

FT result	PCR result		
	**Positive**	**Negative**	
**Positive**	67	4	PPV 94.4 %
**Negative**	32	104	NPV 76.5 %
	Sensitivity 67.7 %	Specificity 96.3 %	

Figure S14. Flow-through (FT) device performance using heat-inactivated primary patient swabs before silver staining step (positive result is test line and control line being visible, with control line decisions guiding by silver (as they were weak and hence not included in main analysis)). A) Results of device performance (hit or miss) as a function of Ct for devices ran alone. Thresholds indicated are the sensitivity as a function of the Ct value; B) Confusion matrices after silver staining. Sensitivity = TP/(TP+FN); Specificity = TN/(TN+FP); PPV = TP/(TP+FP); NPV = TN/(TN+FN). TP = true positive; TN = true negative; FN = false negative; FP = false positive. Larger versions of Figure S14A can be found as Figures S15A and 15B.

Analysis of Patient Samples – Larger Versions of Figure 5A and Figure S14

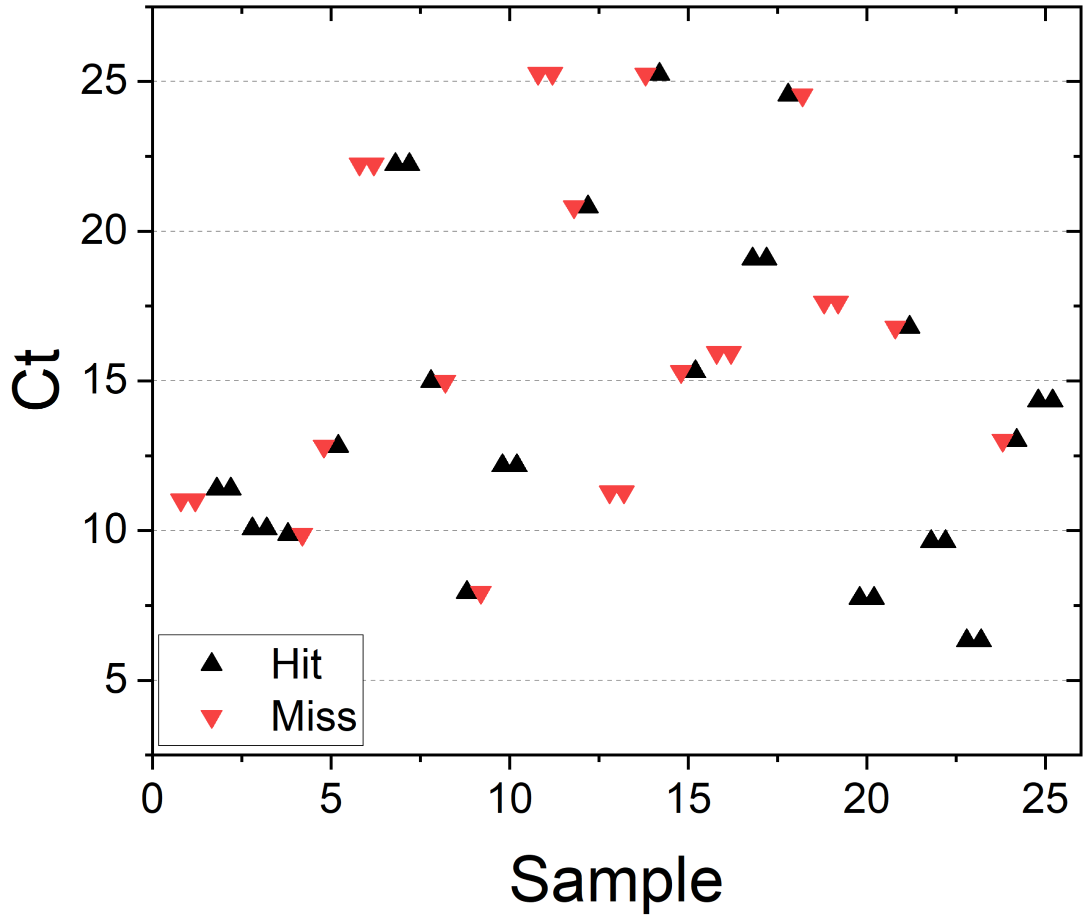

Figure S15A. Flow-through (FT) device performance using heat-inactivated primary patient swabs (1-25) before silver staining step (positive result is test line and control line being visible, with control line decisions guiding by silver (as they were weak and hence not included in main analysis)).

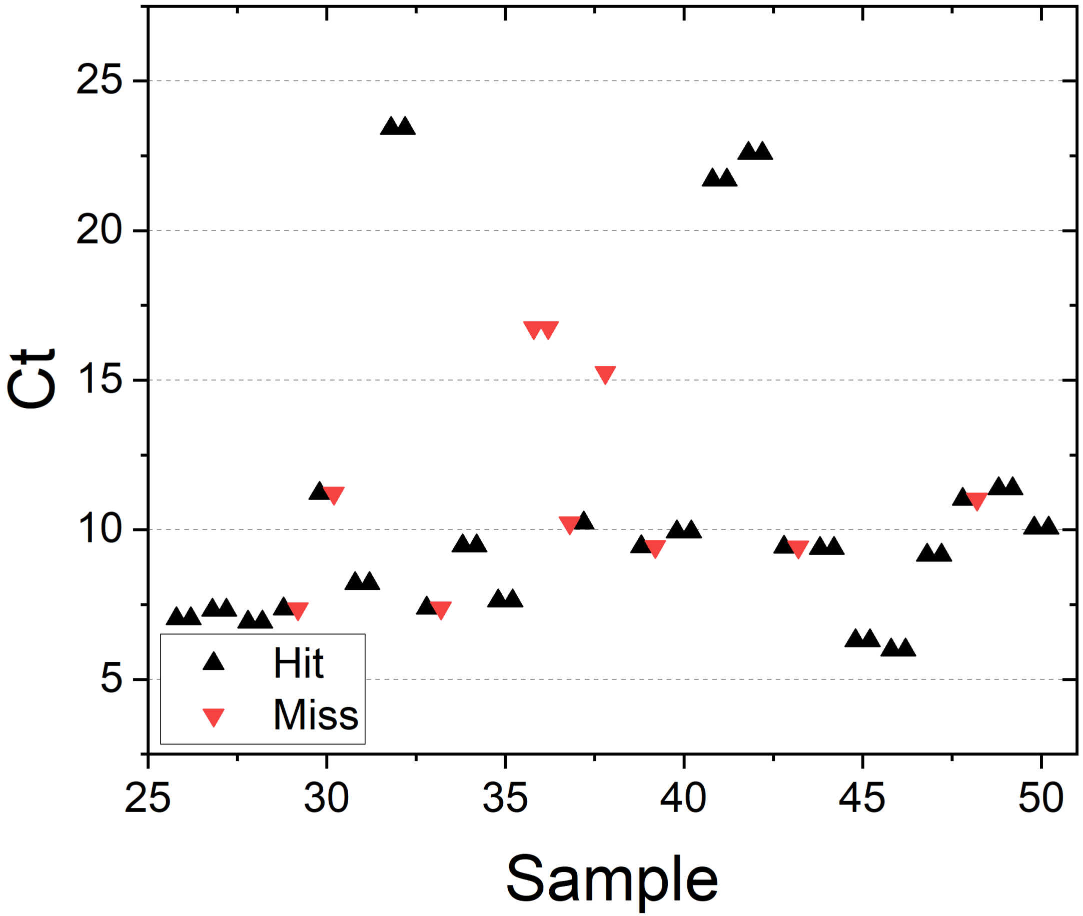

Figure S15B. Flow-through (FT) device performance using heat-inactivated primary patient swabs (26-50) before silver staining step (positive result is test line and control line being visible, with control line decisions guiding by silver (as they were weak and hence not included in main analysis)).

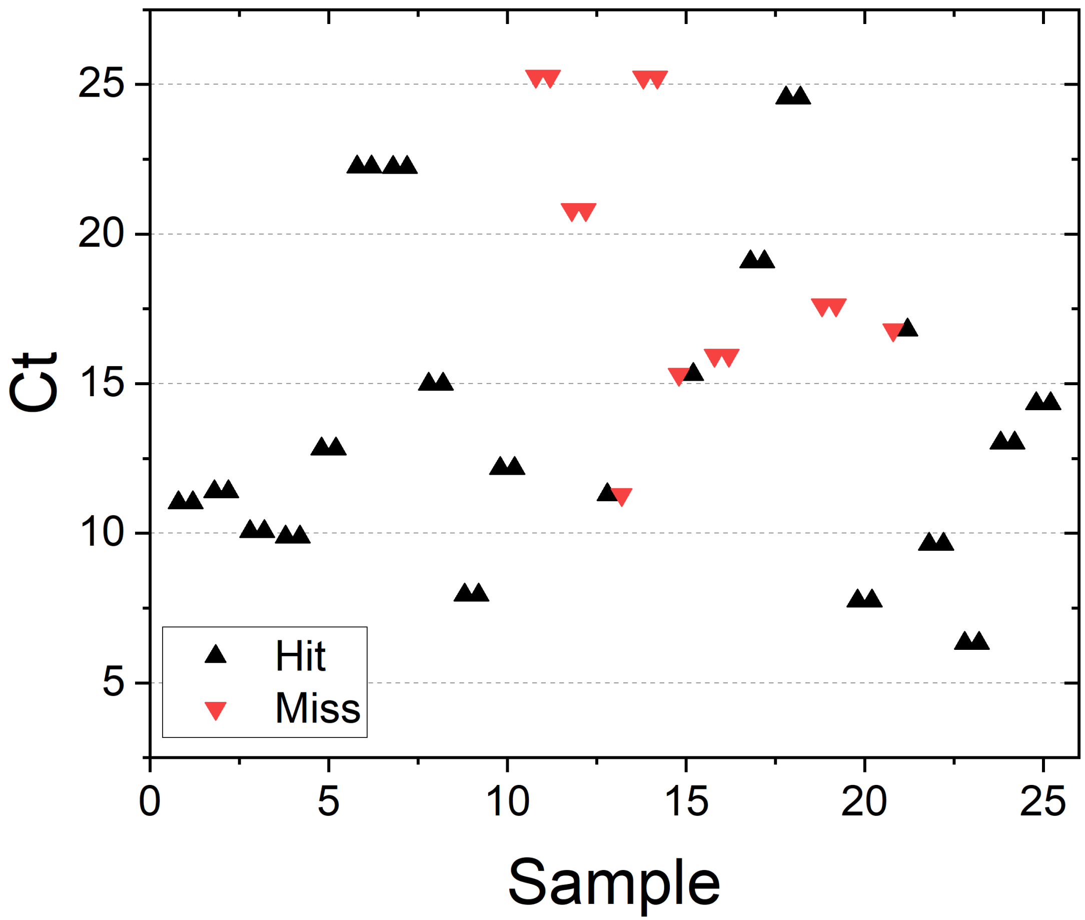

Figure S16A. Flow-through (FT) device performance using heat-inactivated primary patient swabs (1-25) after silver staining step (positive result is test line and control line being visible).

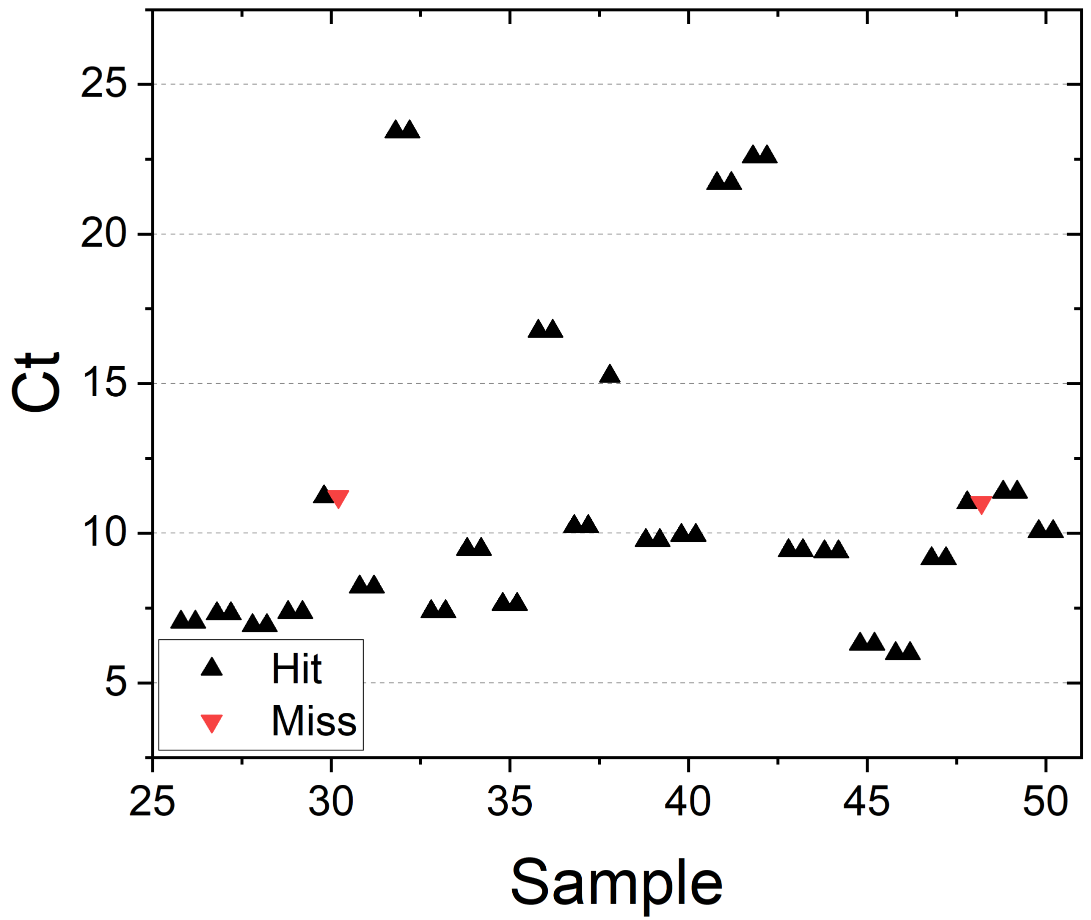

Figure S16B. Flow-through (FT) device performance using heat-inactivated primary patient swabs (26-50) after silver staining step (positive result is test line and control line being visible).

Cassettes from SARS-COV-2 Spike Protein S1, SARS-COV-2 heat-inactivated virus, Influenza viruses and Hemagglutinin Experiments

Tergitol NP-40 (%)	Particles
0	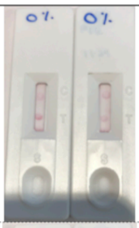
0.1	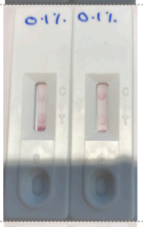
0.5	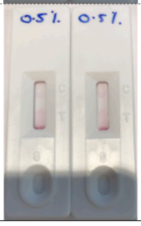

Table S16. Photos of flow-through cassettes versus sample lines of 0.5 mg/mL SC2 S1 protein using buffer solution containing varying concentrations of tergitol NP-40. A control line of 1 mg/mL RCA_{120} was used. Spike protein used was expressed in HEK293 cells, not *E.coli* in this experiment.

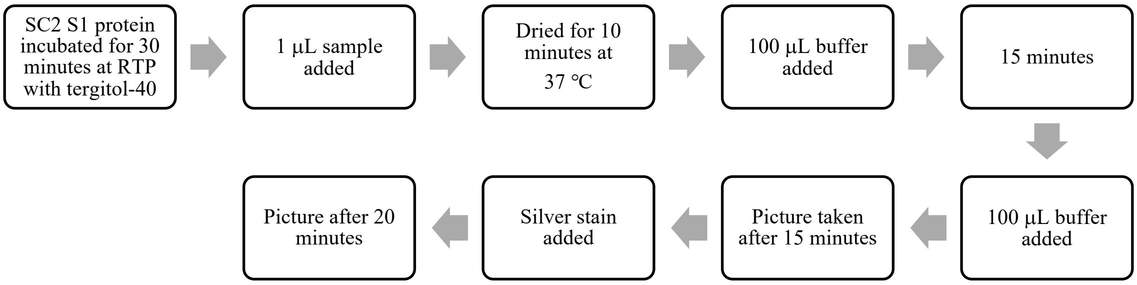

Test line	20 minutes after first buffer addition	20 minutes after second buffer addition	25 minutes after silver stain
Heat inactivated SC2 virus, Preinactivation Titre 1.6 × 10^5 $TCID_{50}$ per mL, 1 spot added	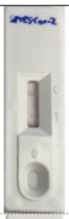	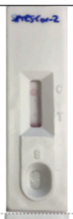	
Heat inactivated SC2 virus, Preinactivation Titre 1.6 × 10^5 $TCID_{50}$ per mL, 2 spots added	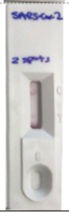	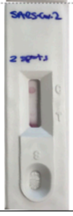	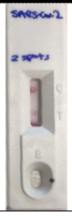

Table S17. Photos of flow-through cassettes versus sample lines of SARS-COV-2 heat-inactivated virus spotted after varying times. A control line of 1 mg/mL RCA_{120} was used. The virus used is SARS-Related Coronavirus 2 (SARS-COV-2), isolate USA-WA1/2020 that has been inactivated by heating to 65°C for 30 minutes.

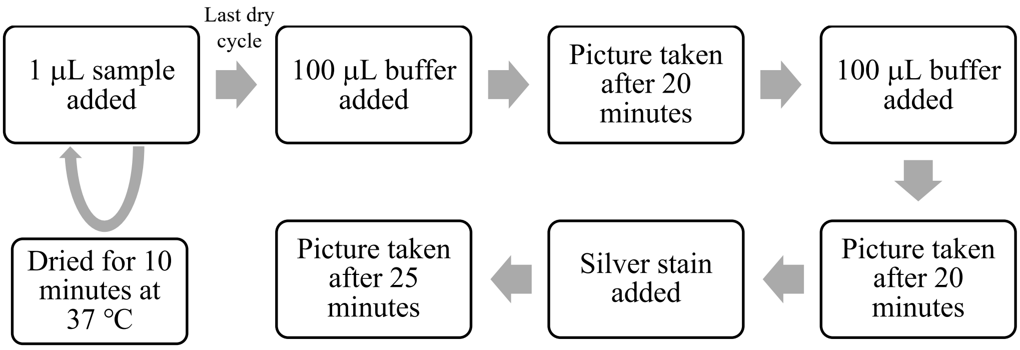

Hemagglutinin	Cassettes
H1	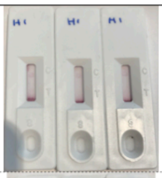
H3	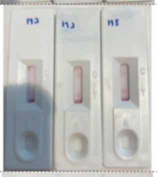
H7 (from H7N3)	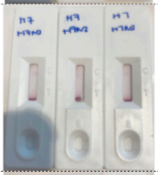
H7 (from H7N9)	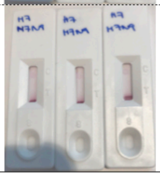

Table S18. Photos of flow-through cassettes versus sample lines of varying hemagglutinins at 0.5 mg/ml. A control line of 1 mg/mL RCA_{120} was used. Hemagglutinins used are as follows; H7 Hemagglutinin (HA) protein from influenza virus (A/Canada/rv444/2004 (H7N3)) – recombinant from Baculovirus, H7 Hemagglutinin (HA) protein from influenza virus (A/Shanghai/1/2013 (H7N9)) – recombinant from Baculovirus, H3 Hemagglutinin (HA) protein from influenza virus (A/New York/55/2004 (H3N2)) – recombinant from Baculovirus (Catalogue number: NR-19241) and H1 Hemagglutinin (HA) protein with C-terminal histidine tag from influenza virus (A/Brisbane/59/2007 (H1N1)) – recombinant from Baculovirus.

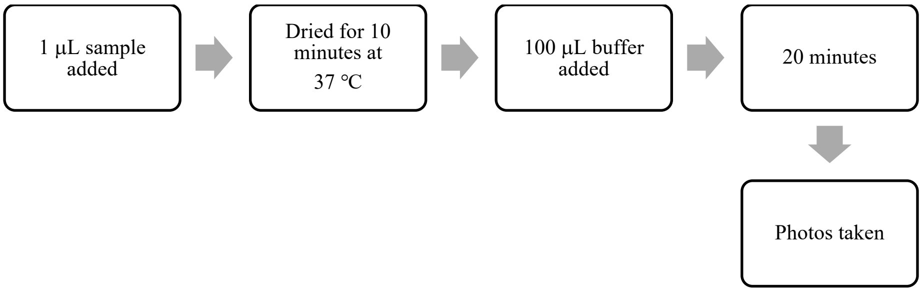

Influenza	After 1st Buffer	After 2nd buffer	After silver stain
H1N1	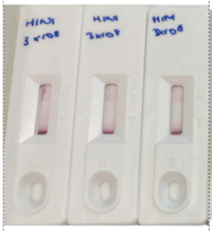	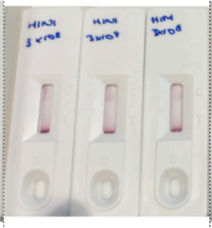	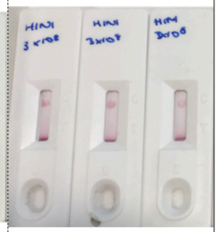
H3N2	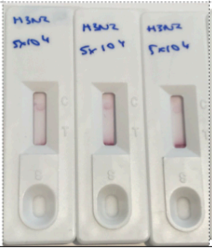	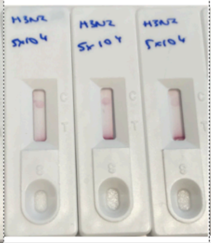	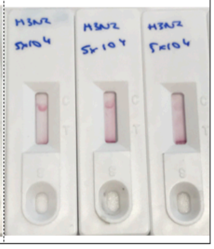

Table S19. Photos of flow-through cassettes versus sample lines of varying influenzas (H1N1 at 3×10^8 $CEID_{50}$ and H3N2 at 5×10^4 $CEID_{50}$). A control line of 1 mg/mL RCA_{120} was used. Influenzas used are as follows; Influenza A virus (A/Brisbane/10/2007 (H3N2)) BPL-inactivated and Influenza A virus (A/Puerto Rico/8/1934 (H1N1)) BPL-inactivated.

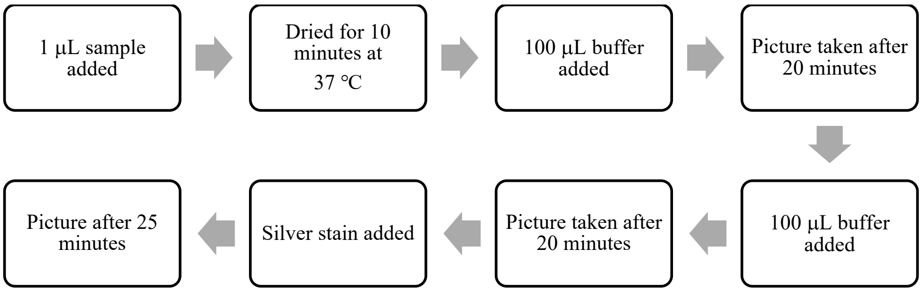

Wild Type<br>Location first identified Wuhan, China	B.1.1.7<br>Location first identified Kent, U.K	B.1.351<br>Location first identified South Africa	P.1<br>Location first identified Brazil

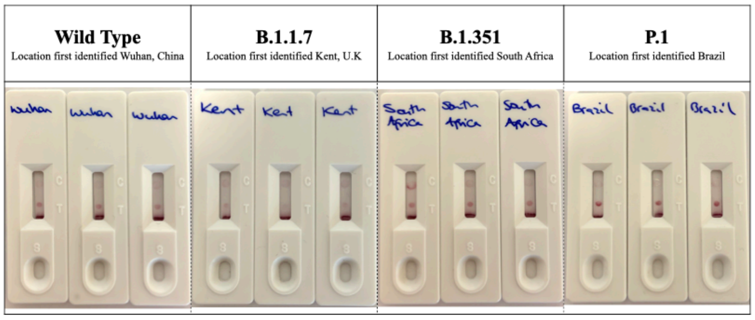

Table S20. Photos of flow-through cassettes versus sample lines of wild type and mutant S1 spike proteins (~0.25 mg/mL). A control line of 1 mg/mL RCA_{120} was used. Details of the spike protein mutants can be found in the section SARS-COV-2 spike protein variants.

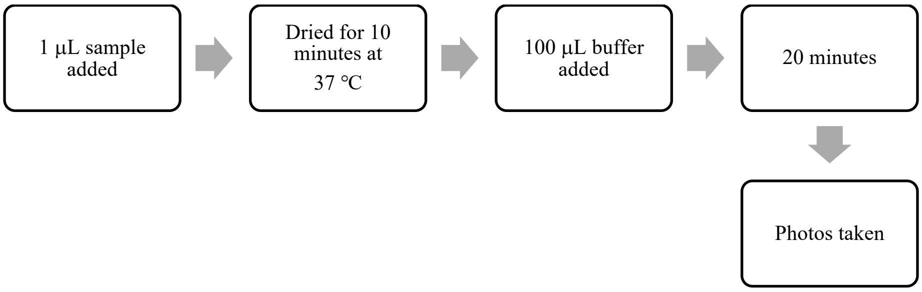

Spike Protein S1	Spike Protein S1 after 60 °C Heat Treatment
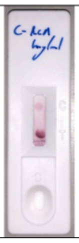	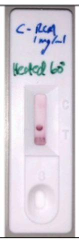

Table S21. Photos of flow-through cassettes versus sample lines of S1 spike protein (~0.25 mg/mL). A control line of 1 mg/mL RCA_{120} was used. Spike Protein after 60 °C heat treatment was heated to 60 °C for 30 minutes before addition as test line.

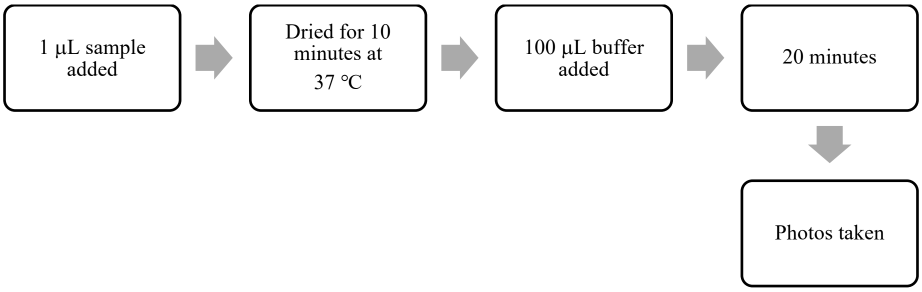

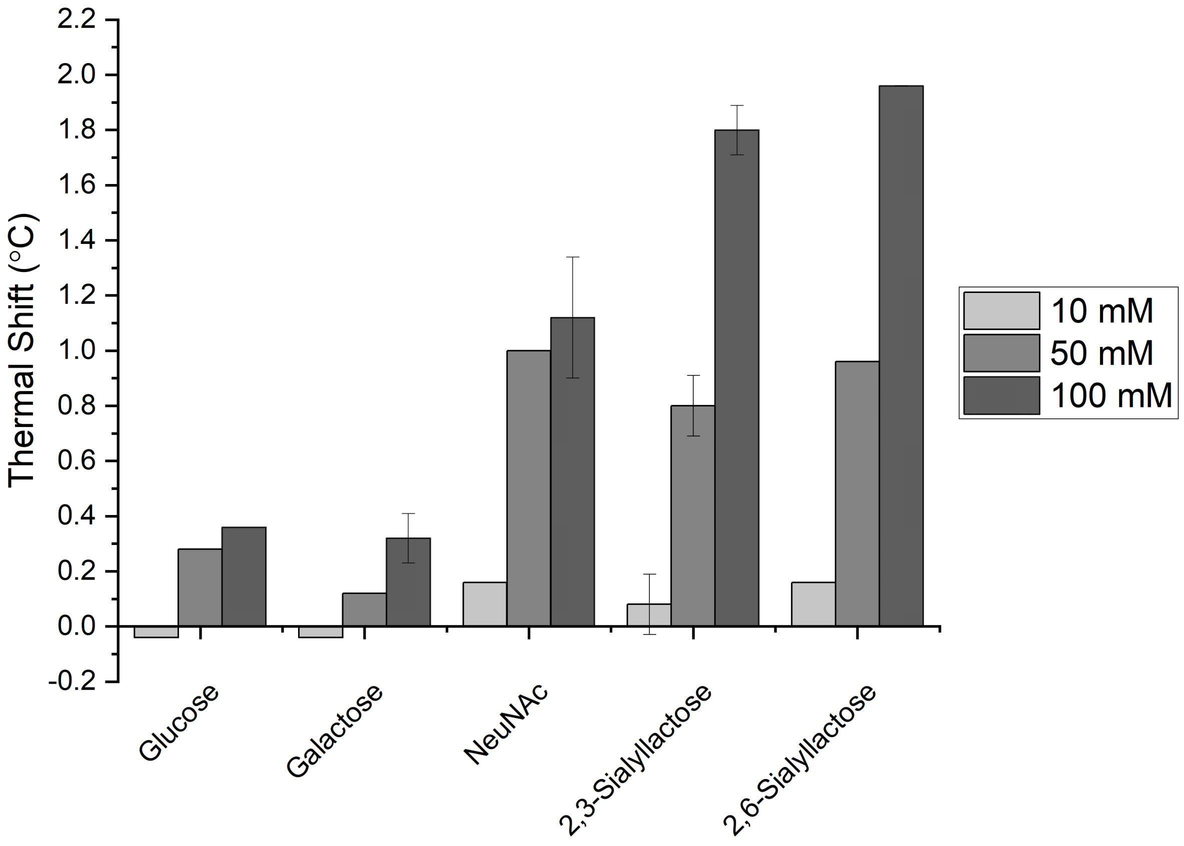

Figure S17. Protein thermal shift assay results plotted as total shift compared to protein alone. Errors bars are SD from a minimum of 5 runs.

References

(1) Baker, A. N.; Richards, S. J.; Guy, C. S.; Congdon, T. R.; Hasan, M.; Zwetsloot, A. J.; Gallo, A.; Lewandowski, J. R.; Stansfeld, P. J.; Straube, A.; Walker, M.; Chessa, S.; Pergolizzi, G.; Dedola, S.; Field, R. A.; Gibson, M. I. The SARS-COV-2 Spike Protein Binds Sialic Acids and Enables Rapid Detection in a Lateral Flow Point of Care Diagnostic Device. *ACS Cent. Sci.* **2020**, *6* (11), 2046–2052.

(2) Abbott Molecular Inc. *Abbot RealTime SARS-CoV-2 Instructions for Use (Ref: 09N77-095)*; 2020.

(3) Bastús, N. G.; Comenge, J.; Puntes, V. Kinetically Controlled Seeded Growth Synthesis of Citrate-Stabilized Gold Nanoparticles of up to 200 Nm: Size Focusing versus Ostwald Ripening. *Langmuir* **2011**, *27* (17), 11098–11105.

(4) Haiss, W.; Thanh, N. T. K.; Aveyard, J.; Fernig, D. G. Determination of Size and Concentration of Gold Nanoparticles from UV - Vis Spectra. *Anal. Chem.* **2007**, *79* (11), 4215–4221.
